# Novel gemini cationic thiazole-based surfactants as carbon steel corrosion inhibitors in 1 M HCl using experimental and theoretical tools

**DOI:** 10.1038/s41598-025-02173-x

**Published:** 2025-05-20

**Authors:** Samir A. Abd El-Maksoud, Mohamed A. Migahed, Mahmoud M. Gouda, Farid I. El-Dossoki

**Affiliations:** 1 Chemistry Department, Faculty of Science, Port-Said University, Port-Said, Egypt; 2 Petroleum Applications Department, Egyptian Petroleum Research Institute (EPRI), Cairo, Egypt

**Keywords:** Inhibitors, Corrosion, Adsorption, Surfactant, Synergistic, Physical chemistry, Thermodynamics

## Abstract

This paper examines two novel Gemini cationic surfactants based on thiazole derivatives (TAC) as anticorrosion compounds for carbon steel in 1 M HCl. This task was achieved using a diversity of tools, comprising mass loss (ML), potentiodynamic polarization (PP), electrochemical impedance spectroscopy (EIS), scanning electron (SEM) microscope, energy dispersive X-ray (EDX), Fourier transform infrared spectroscopy (FTIR), and computational density functional (DFT) theory. The shift in corrosion potential revealed the compounds’ efficacy as cathodic inhibitors. The impedance measurement confirmed that a shielding film had formed on the carbon steel. The inhibitory action was found to be increased with increasing the inhibitor concentration which reached 79% for TAC 6 and 87% for TAC 18 at 50 ppm, while it slightly decreased with raising the temperature from 303 to 323 K. The mechanism of adsorption minds the Langmuir adsorption isotherm. The mixed physical and chemical adsorption of the inhibitors on the steel surface was confirmed by thermodynamics and kinetic characteristics. Electrochemical techniques examined the synergism inhibition of both inhibitors in the presence of inorganic salts at 303 K which indicated to follow CuCl_2_ > MnCl_2_ > CoCl_2_. The synergetic inhibition of both inhibitors in the presence of CuCl_2_ reached 96% for TAC 6 and 97% for TAC 18. Both inhibitors in the presence of salts were changed to act as mixed-type inhibitors.

## Introduction

It is well known that HCl solution is considered one of the mineral acids utilized in many industrial procedures, which include pickling, acid cleaning and descaling, petrochemical manufacturing, oil, and natural gas extraction, especially for old wells, and finally, geothermal water extraction^1–7^ Unfortunately, when we use HCl solution in these applications, carbon steel alloys can be corroded, which is a tremendous waste of money and resources^8–10^. For this reason, special attention must be applied to prevent steel degradation to reduce the amount of harmful metal ions produced from steel degradation entering the ecosystem^11,12^. Corrosion avoidance methods prefer using chemical compounds with an environmentally low impact.

One of the most practical ways to prevent metals from corroding is to utilize Gemini cationic surfactant molecules as corrosion inhibitors, gaining popularity^13–22^. According to existing research, organic inhibitors work by adsorption and form a protective shield around the metal^23,24^.

Organic molecules having multiple bonds that are considered adsorption centers or adsorption centers such as phosphorus, sulfur, nitrogen, and oxygen heteroatoms with high electron density, are effective corrosion inhibitors^25–32^. Doubling the hydrophilic head groups and hydrophobic tails increased the efficiency of surfactants in inhibiting steel breakdown in an acidic media^33,34^.

The efficiency of inhibition of Gemini cationic surfactants for carbon steel in an acidic medium is related to surfactant concentrations, media temperature, immersion time, the structure of surfactants, and the synergetic effect of additional salts^35–43^. The derivatives of thiazole have been studied before as corrosion inhibitors and confirmed higher inhibition efficiencies^44–48^.

This study explores the recently synthesized Gemini cationic surfactants (TAC 6 and TAC 18) as non-toxic corrosion inhibitors prepared in our laboratory^49^. These findings offer a cost-effective, eco-friendly solution for prolonging petroleum infrastructure lifespan, reducing maintenance costs, improving pipeline safety, acidizing treatments, and well-stimulation processes^7,50,51^. Unlike conventional research that examines surfactant inhibitors alone, this work investigates their synergistic effect with inorganic salts (CoCl₂, MnCl₂, CuCl₂).

This paper focuses on the inhibition performance of a TAC series with diverse hydrocarbon chain lengths (*n* = 6 and 18) by ML, PP, and EIS measurements. Surface examination tools (SEM, EDX, FTIR) are used and confirmed by computational quantum calculations (DFT).

## Experimental

### Working electrode Preparation

The carbon steel specimen used is composed of C (0.14%), Cr (0.10%), Ni (0.10%), Si (0.024%), Mn (0.05%), P (0.05%), and Fe (balance). The copper rod responsible for electrical conductivity was fixed to a carbon steel sheet with an area of 1 cm^2^ which was exposed to a corrosive medium. The pieces were inserted into a Teflon cover and fitted with epoxy resin. The carbon steel surface was polished with different grades (800-1000-1200) of emery paper to measure the required smoothness. The electrode surface was washed and degreased with bi-distilled water and acetone, respectively, and then dried with filter paper.

### Chemicals

The studied substances were obtained from Sigma-Aldrich, a global firm with headquarters in the United States, and were of a high purity grade, meaning further purification was not necessary: hydrochloric acid (37%), and acetone (99.9%). Chloride salts of Co, Mn, and Cu with a purity of 98% were used.

### Test solutions

The corrosion medium was carried out in HCl deprived of and using diverse concentrations of TAC 6 and TAC 18 Gemini cationic surfactants; the molecular structure is shown in Structure 1. Terephthalaldehyde was refluxed with thiazol-2-amine in ethanol for 12 h in the presence of p-toluene sulfonic acid as a dehydrating agent. The intermediate was further refluxed with different alkyl halides (C₆H₁₃ and C₁₈H₃₇) in acetone. The resulting compounds were purified by filtration, recrystallization, washing, and vacuum drying at 60 °C. The final products were TAC 6 and TAC 18, corresponding to hexyl and octadecyl thiazolium bromide derivatives^49^. The corrosive solutions were prepared by fresh diluting a high analytical grade of concentrated hydrochloric acid with doubly distilled water. The concentration of inhibitors used in this study varied from 5 to 50 ppm. Salt solutions are prepared at several concentrations from 10 to 90 ppm. The temperature was controlled thermostatically at different temperatures ranging from 30 °C to 50 ℃.

**Structure 1:**
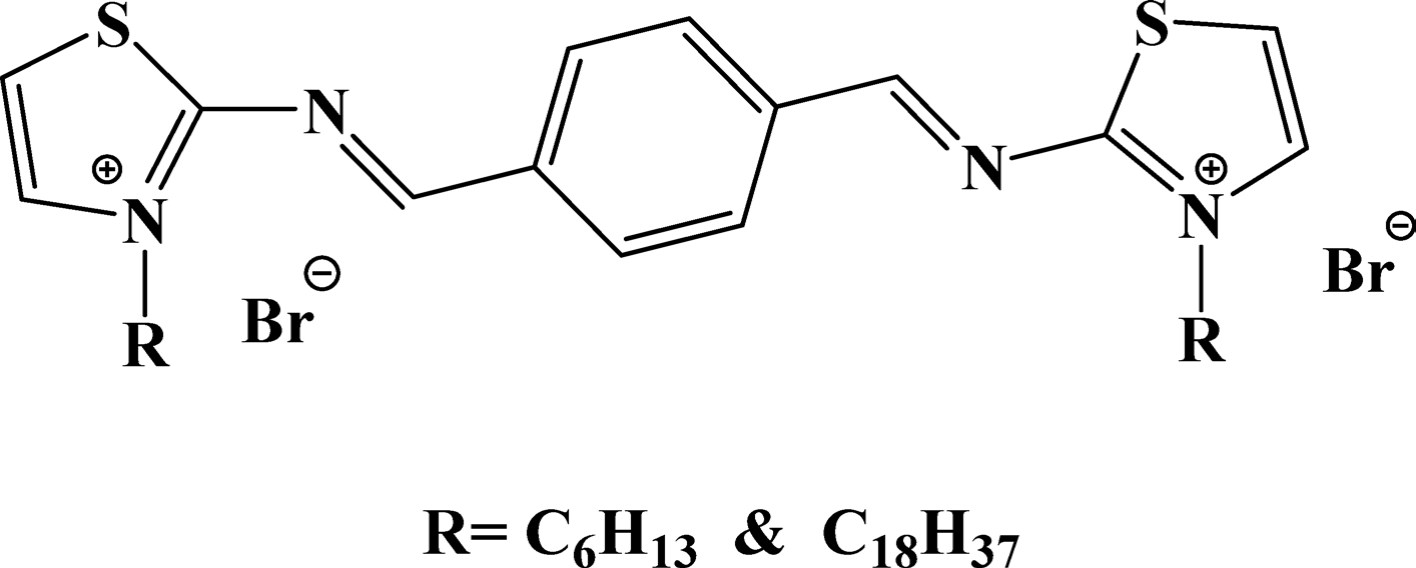
The molecular structure of TAC Gemini cationicsurfactants.

### Mass loss quantification

Carbon steel specimens with dimensions of $\:2.0\times\:2.0\times\:0.2$ cm were polished with varied emery papers with different grades of 800, 1000, and 1200, washed, degreased, and dried with filter paper. The prepared steel specimens were then accurately weighed by using a USS-DBS10 Digital Analytical Balance Scales, readability 0.1 mg, U.S. Solid, United States. The specimens were submerged in hydrochloric acid without and with diverse concentrations of Gemini cationic surfactants for 48 h. Once every eight hours, samples were detached from the corrosive solution, scrubbed, dried, and reweighed three times, and then the average weight was calculated.

### Electrochemical techniques

Electrochemical measurements were done by using a high-performance potentiostat/galvanostat /ZRA, Reference 3000 with Echem Analyst 2 software, Gamry Instruments, United States. A cell with three electrodes was used. Calomel and platinum electrodes are used as reference and auxiliary electrodes, respectively. The working electrode was submerged in a corrosive solution for 30 min to ensure the establishment of a steady-state potential ($\:{E}_{ocp}$), then other measurements were done. The EIS technique was performed around the frequency range of 100–0.0002 kHz with an amplitude of 10 mV at an ($\:{E}_{ocp}$). By fitting the experimental data extracted from Echem Analyst software according to a different equivalent circuit, the impedance can be calculated. The potentiodynamic polarization test was indicated by sweeping the potential range of $\:\pm\:250$ mV from $\:{E}_{ocp}$, starting from the cathodic potential to the anode site with a sweep rate of 1 mV s^−1^.

### Surface examination

The carbon steel surface was examined without and with 50 ppm of TAC 6 Gemini cationic surfactant at two different temperatures. SEM was used to scan the surface morphology after 24 h of immersion. The elements on the surface carbon steel were indicated by energy dispersive X-ray (EDX) on the same specimens used in SEM examination. Fourier transform infrared (FT-IR) spectroscopy is used to confirm the functional groups adsorbed from TAC 6 surfactant on a carbon steel surface after 8 h of immersion.

### Computational DFT technique

Quantum simulations were carried out via Gaussian software version 9.0 to examine the impact of molecular structure on inhibition efficiency. Complete geometry optimization was used throughout all calculations, and the hybrid B3LYP functional level with a higher basis set, designated by 6–31 G, was used (d, p). Gauss View was used to evaluate the energy of the highest occupied molecular orbital (E_HOMO_), the energy of the lowest unoccupied molecular orbital (E_LUMO_), and the energy gap (ΔE).

**Fig. 1:**
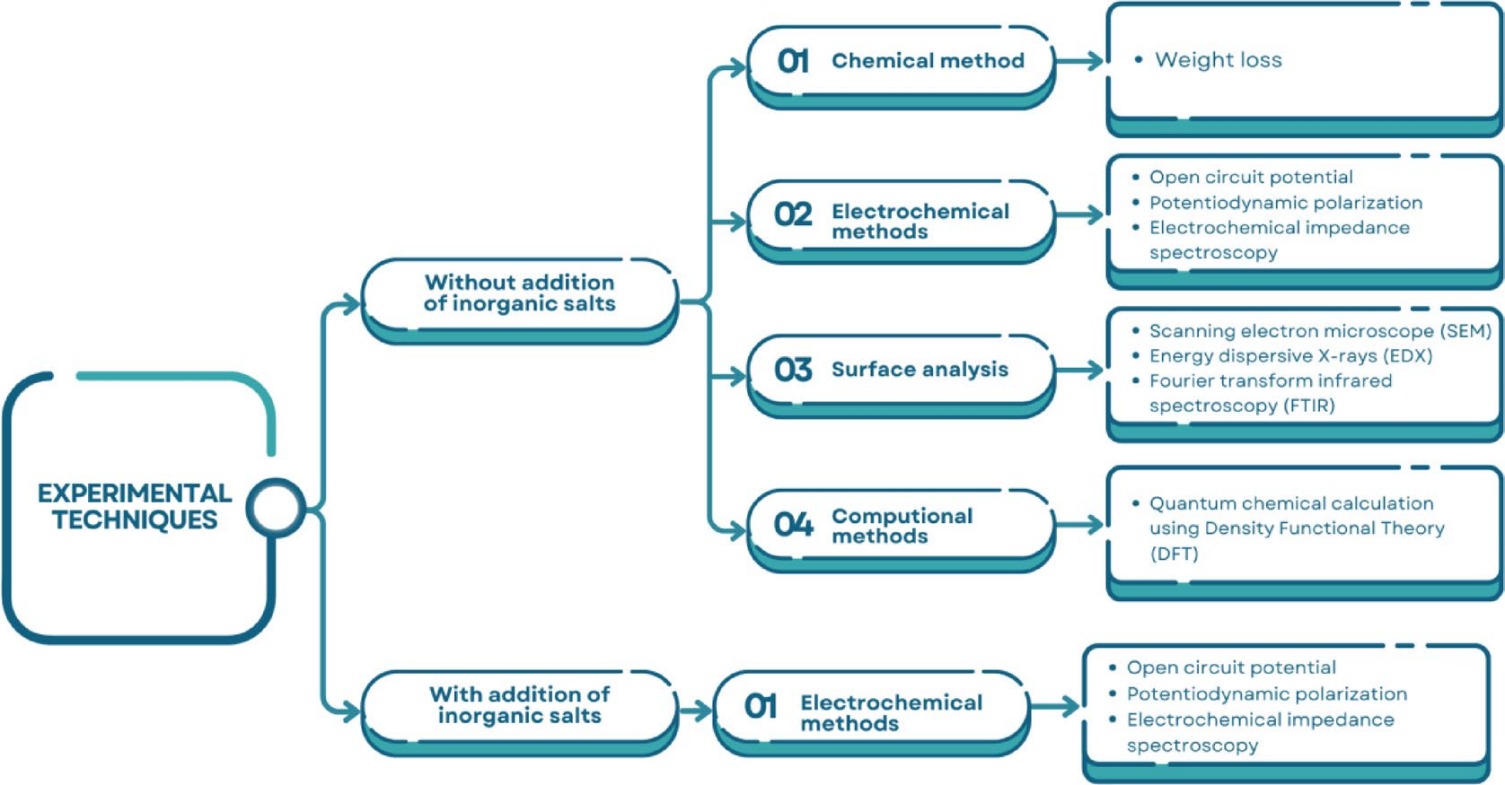
A schematic representation of the experimental techniques used to evaluate the corrosion inhibition performance of Gemini cationic surfactants for carbon steel in 1 M HCl, both in the absence and presence of inorganic salts. The methods include weight loss measurements, electrochemical techniques (open circuit potential, potentiodynamic polarization, electrochemical impedance spectroscopy), surface analysis (SEM, EDX, FTIR), and computational studies (DFT).

The experimental routes of all techniques used in this examination are shown in Fig. 1.

## Results and discussion

### Mass loss quantification

#### Effect of immersion time

**Fig. 2:**
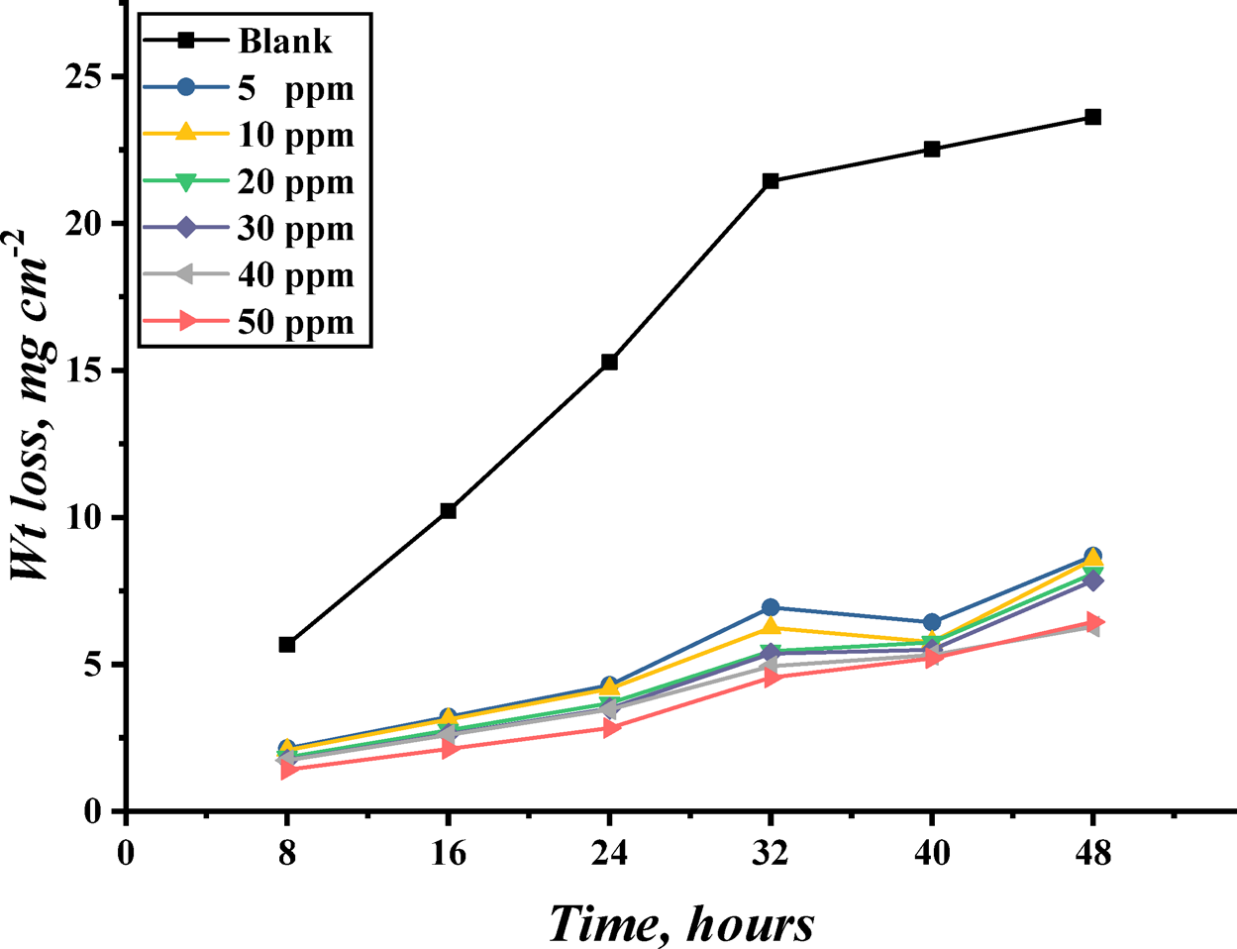
Mass loss of carbon steel in 1 M HCl over immersion time with various concentrations of TAC 6 inhibitor at 303 K.

The data collected from mass loss measurements were used to calculate the corrosion rate of carbon steel specimens in a 1 M hydrochloric acid solution in the absence and presence of different concentrations of TAC 6 and TAC 18. The efficiencies of inhibitions were calculated according to Eq. 1.

1 $$\:\text{I}\text{E}\:\text{\%}\:=\left[1-\frac{{\text{W}}_{\text{i}\text{n}\text{h}.}}{{\text{W}}_{\text{b}}}\right]\times\:100$$

where $\:\frac{{\text{W}}_{\text{i}\text{n}\text{h}.}}{{\text{W}}_{\text{b}}}$ is the ratio between the mass loss in the inhibited solution and the blank solution. Table 1 includes data indicated from mass loss, corrosion rate ($\:{K}_{corr}$), surface coverage (θ), and inhibition efficiencies (IE%). The corrosion rate decreases with the increase of the inhibitor concentration, consequently, the inhibition efficiency of the inhibitors increases, indicating that the increase in the inhibitor concentration indicates an increase in the capacity for adsorption^52^. The inhibition efficiency of TAC 18 is higher than TAC 6. The hydrocarbon chains play a vital role in hydrophobic interaction and shielding on the steel surface to create a protective layer on the steel surface, making it difficult for corrosive chloride ions and water molecules to adsorb on the steel surface, thereby inhibiting steel dissolution^53^. The mass loss of carbon steel specimens through the immersion times is shown in Fig. 2.

**Table 1:** Values of mass loss, corrosion rate ($\:{K}_{corr}$), surface coverage (θ), and Inhibition efficiencies (ɳ%) for both inhibitors at 25 °C after 32 h.

Solution	Conc. (ppm)	Mass loss (mg cm^− 2^)	$k_{corr}$ (mg cm^− 2^ h^− 1^)	θ	IE (%)
Blank	0	21.43	0.670	–	–
TAC 6	5	6.938	0.217	0.676	67.63
	10	6.243	0.195	0.709	70.87
	20	5.445	0.170	0.746	74.59
	30	5.369	0.168	0.750	74.95
	40	4.936	0.154	0.770	76.97
	50	4.547	0.142	0.788	78.79
TAC 18	5	4.889	0.153	0.772	77.19
	10	4.538	0.142	0.788	78.82
	20	3.478	0.109	0.838	83.77
	30	3.405	0.106	0.841	84.11
	40	2.808	0.088	0.869	86.90
	50	2.688	0.084	0.875	87.46

#### Effect of temperature

**Fig. 3:**
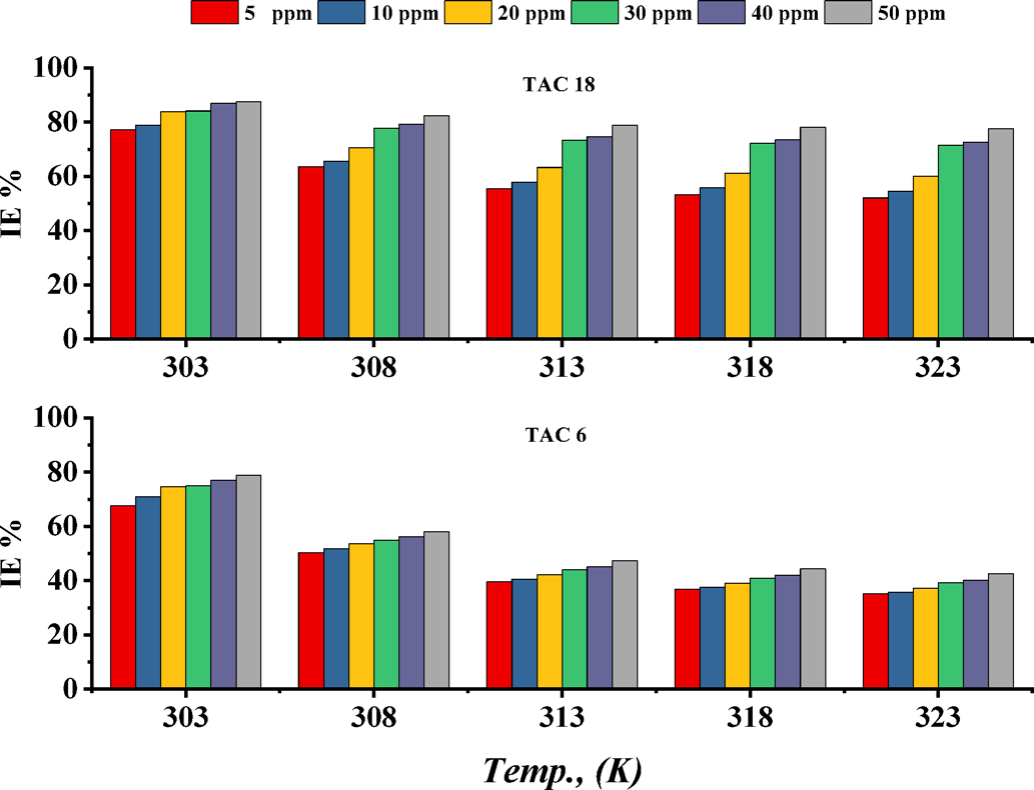
The inhibition efficiencies of inhibitors TAC 6 and TAC 18 with various concentrations (5–50 ppm) under the influence of different temperatures 303–323 K.

The effect of temperature on the inhibition efficiencies of TAC 6 and TAC 18 on specimens in a 1 M HCl solution was studied at various temperatures in the range between 30⁰C and 50⁰C. The correlation between temperature and inhibition efficiencies was detected in Fig. 3. The inhibition efficiencies decreased with the increase in temperature^54^. At low temperatures, the heteroatoms nitrogen and sulfur, as well as the lone pairs of electrons in a benzene ring, create an electrostatic attraction to the steel surface, forming a film on the surface^55^. As the temperature increases, the chloride ions from the HCl solution contest to adsorb on the surface, decreasing the surface coverage of inhibitors and decreasing inhibition efficiency^56^.

#### Kinetic and thermodynamic parameters

The activation parameters of the corrosion reaction between carbon steel specimens and 1 M HCl solution were calculated from the Arrhenius relationship (Eq. 2)^57^ and transition state (Eq. 3)^58^ as follows:

2 $$\:log{K}_{corr}=\frac{{-E}_{a}^{*}}{2.303\:RT}+log\:A$$

3 $$\:log\frac{{K}_{corr}}{T}=log\frac{R}{N.h}+\frac{{\varDelta\:S}^{*}}{2.303\:R}-\frac{{\varDelta\:H}^{*}}{2.303\:RT}$$

**Fig. 4:**
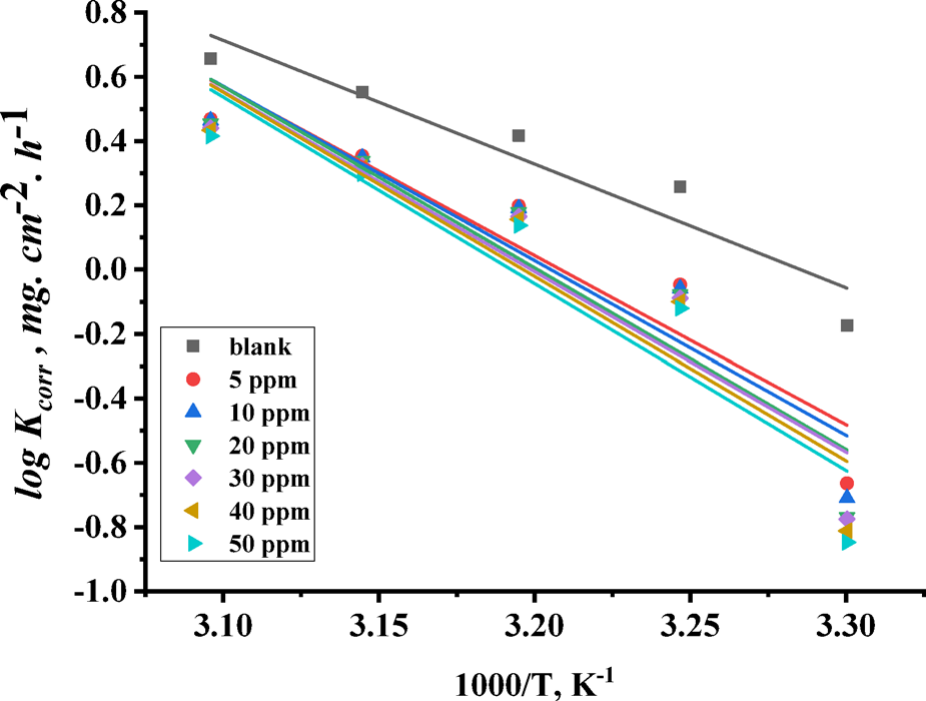
The Arrhenius relationship of carbon steel in 1 M HCl in the absence and presence of different concentrations (5 to 50 ppm) TAC 6.

**Fig. 5:**
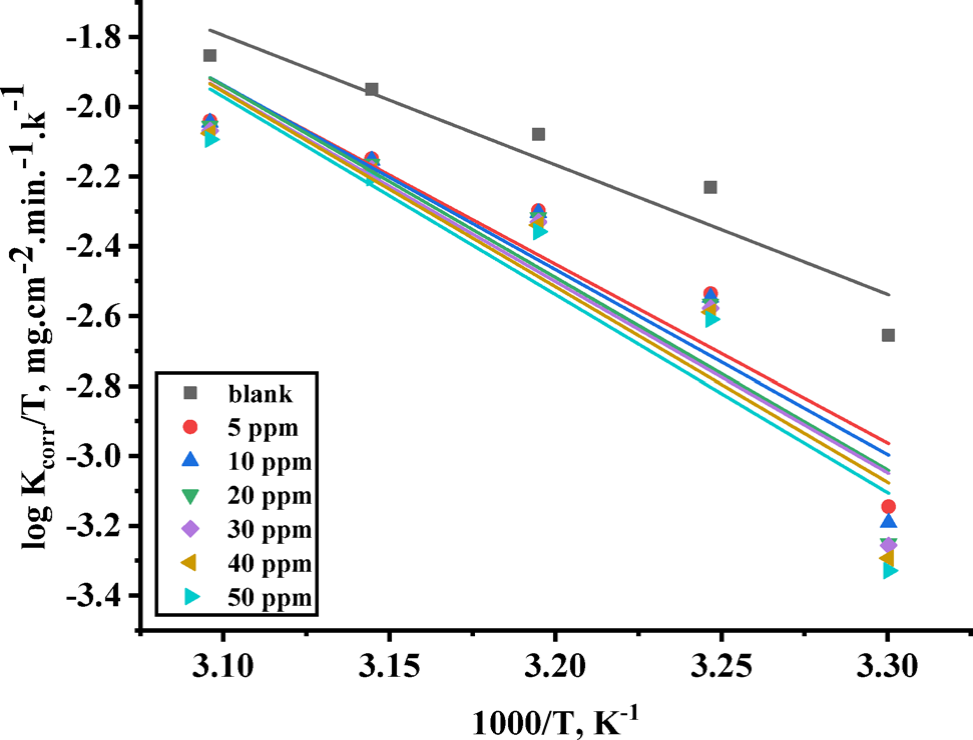
$\:log{(K}_{corr}/T)\:$ was plotted against $\:1000/T$ of C-steel in 1 M HCl in the absence and presence of different concentration (5 to 50 ppm) TAC 6.

$\:{E}_{a}^{*}$ is the activation energy, A is the Arrhenius constant, R is the universal gas constant, T is the absolute temperature, h is Planck’s constant, N is the Avogadro’s number, $\:{\varDelta\:S}^{*}$ is the entropy of activation and $\:{\varDelta\:H}^{*}$is the enthalpy of activation. By determining the slope of the relationship between $\:log{(K}_{corr})$ and $\:(1/T)$, activation energy ($\:{E}_{a}^{*}$) can be calculated as shown in Fig. 4. By plotting the relation between $\:\text{l}\text{o}\text{g}{(K}_{corr}/T)$ and $\:(1/T)$, the enthalpy of activation ($\:{\varDelta\:H}^{*}$) can be calculated from their slope, and the entropy of activation ($\:{\varDelta\:S}^{*}$) can be detected from the intercepts as shown in Fig. 5. The activation parameters are summarized in Table 2.

The higher value of activation energy ($\:{E}_{a}^{*}$) in the case of the presence of inhibitors is indicated by confirming the adsorption on the steel surface and decreasing the corrosion rate. The positive magnitude of enthalpy ($\:{\varDelta\:H}^{*}$) describes the corrosion process as an endothermic reaction. The high enthalpy values in the presence of different concentrations of TAC 6 and TAC 18 confirm the need for more energy to overcome the activation energy of the corrosion reaction. The fluctuations of entropy values indicate that the orderliness decreases during transitioning from reactant to activated complex, while at high concentrations, it tends to order in association more than dissociation^59,60^.

**Table 2:** Activation parameters of carbon steel corrosion in 1 M HCl without and with different concentrations at different temperatures.

Soln. Name	Conc. ppm	$\:{\varvec{E}}_{\varvec{a}}^{*}$ kJ mol^− 1^	*R* ^2^	$\:\varDelta\:{\varvec{H}}^{*}$ KJ mol^− 1^	$\:\varDelta\:{\varvec{S}}^{*}$ J mol^− 1^K^− 1^	*R* ^2^
Blank	**0**	32.0	0.95	30.9	-11.50	0.94
TAC 6	**5**	43.6	0.92	42.5	68.90	0.92
	**10**	45.1	0.91	44.0	79.40	0.91
	**20**	46.6	0.90	45.7	91.90	0.90
	**30**	47.0	0.90	45.5	89.70	0.90
	**40**	47.6	0.90	46.5	97.00	0.90
	**50**	48.2	0.90	47.1	101.0	0.90
TAC 18	**5**	44.4	0.91	43.3	71.80	0.91
	**10**	46.4	0.90	43.6	73.70	0.90
	**20**	46.8	0.91	45.7	87.60	0.91
	**30**	48.6	0.92	40.8	49.60	0.92
	**40**	49.1	0.91	43.1	46.10	0.91
	**50**	49.6	0.91	40.8	47.40	0.91

#### Adsorption isotherms

**Fig. 6:**
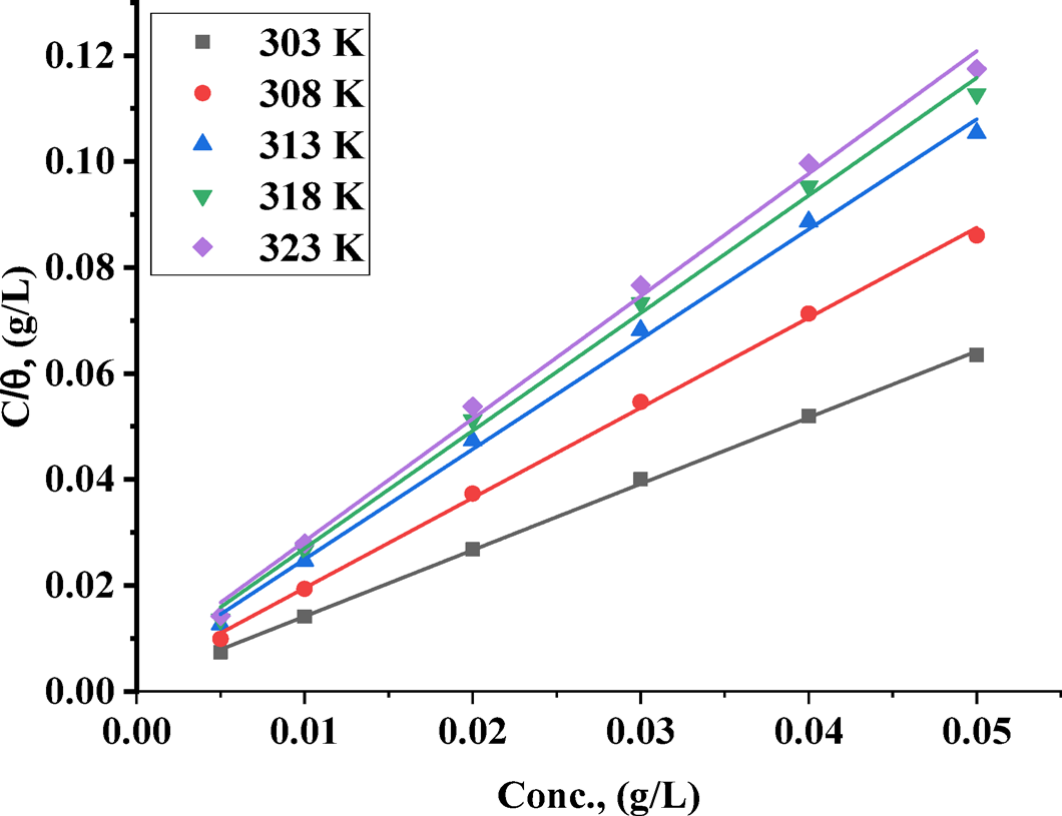
Langmuir adsorption isotherm model for carbon steel surface with TAC 6 inhibitor at different temperatures.

Adsorption isotherms describe the adsorption of the TAC 6 onto the surface of carbon steel during the corrosion inhibition process, which provides insights into the nature and strength of interactions between the inhibitor molecules and the steel surface. Various adsorption isotherms were employed to indicate the best-fitting isotherm that explains the best mechanism of adsorption of TAC on the surface^61,62^. The relationship between concentrations of inhibitors and surface coverage on steel surfaces at different temperatures was plotted to determine the best straight-line fitting for the examined inhibitors. The adsorption isotherm relations are summarized in Table 3. The best fitting obeys the Langmuir isotherm (R^2^ = 1) as shown in Fig. 6 and given by Eq. 4.

4 $$\:\frac{{C}_{\text{i}\text{n}\text{h}}}{\theta\:}=\frac{1}{{K}_{\text{a}\text{d}\text{s}}}+{C}_{\text{i}\text{n}\text{h}}$$

θ is the surface coverage of the steel surface, $\:{C}_{\text{i}\text{n}\text{h}}$ is the concentration of surfactants (mol L^− 1^), and $\:{K}_{\text{a}\text{d}\text{s}}$ is the adsorption constant M^− 1^ which is used to calculate Gibbs free energy of adsorption ($\:{\varDelta\:G}_{ads}^{^\circ\:}$) according to Eq. 5

5 $$\:{K}_{ads}=\frac{1}{55.5}\:\text{e}\text{x}\text{p}\:\left[\frac{{-\varDelta\:G}_{ads}^{^\circ\:}}{RT}\right]$$

The value 55.5 is considered the water concentration in an acid solution^63^. The negativity of Gibbs’s free energy of adsorption ($\:{\varDelta\:G}_{ads}^{^\circ\:}$) confirms the spontaneous formation of an adsorbed layer of both examined surfactants on the steel surface^64^. The value of Gibbs free energy of adsorption is considered an indication of the adsorption kind formed on the surface, which up to -20 kJ/mol indicates the physical adsorption, while higher than − 40 kJ/mol indicates the formation of chemical adsorption^65^. The calculated Gibbs free energy of adsorption ($\:{\varDelta\:G}_{ads}^{^\circ\:}$) confirms the mixed physical and chemical adsorption of both examined inhibitors, TAC 6 and TAC 18 on the steel surface, as summarized in Table 3^66^.

**Table 3:** The square correlation values of different adsorption isotherms at different temperatures.

Adsorption isotherm	Frumkim	El-Awady	Temkin	Freundlish	Langmuir
R^2^	0.90–0.95	0.88–0.92	0.91–0.98	0.87–0.97	0.99-1.00

The variation in the thermodynamic parameters, including enthalpy ($\:{\varDelta\:\varvec{H}}_{\varvec{a}\varvec{d}\varvec{s}}^{^\circ\:}$) and entropy ($\:{\varDelta\:\varvec{S}}_{\varvec{a}\varvec{d}\varvec{s}}^{^\circ\:}$) for adsorption of both examined inhibitors on the steel surface was calculated from integrated Van’t Hoff Eq. 6^67^ or Gibbs-Helmholtz Eq. 7^68^.

6 $$\:{\varDelta\:G}_{ads}^{^\circ\:}={\varDelta\:H}_{ads}^{^\circ\:}-T{\varDelta\:S}_{ads}^{^\circ\:}$$

7 $$\:\text{l}\text{o}\text{g}{K}_{ads}=\frac{{-\varDelta\:H}_{ads}^{^\circ\:}}{2.303\:RT}+constant$$

**Fig. 7:**
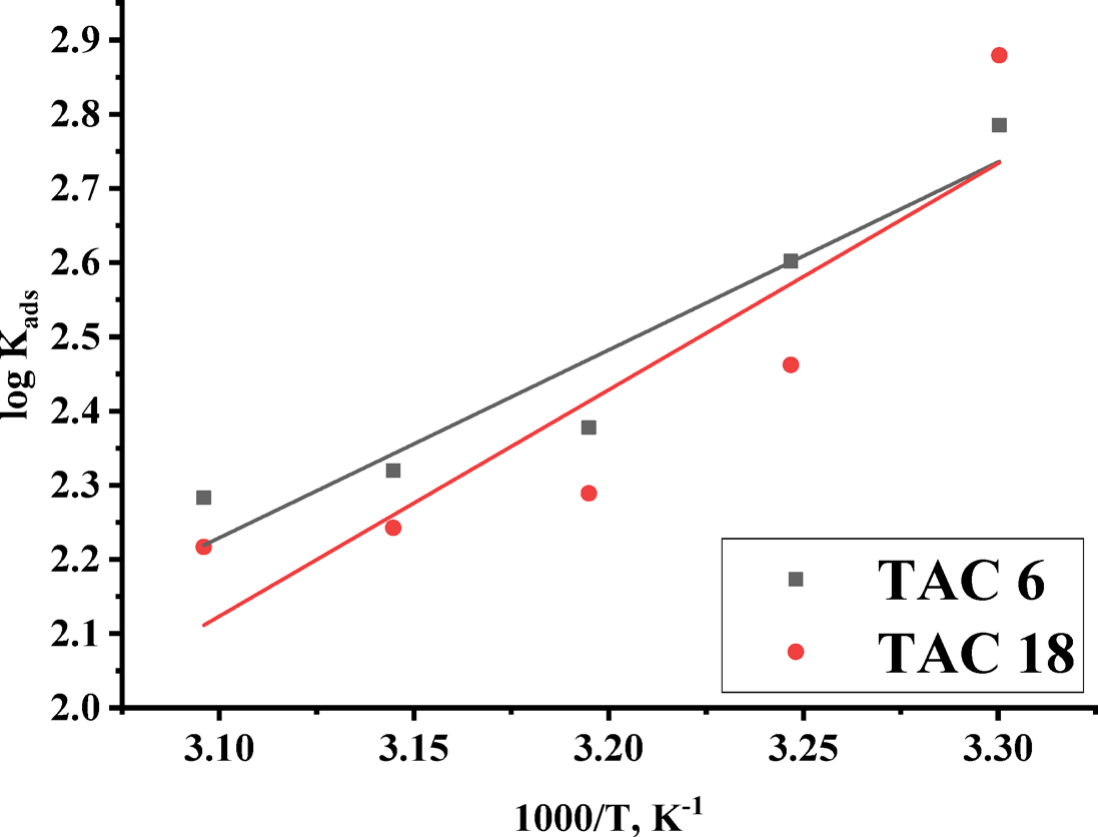
log $\:{K}_{ads}$ was plotted against 1000/T (K) for both inhibitors

By plotting the relationship between $\:\text{l}\text{o}\text{g}{K}_{ads}$ and $\:1/T$, Enthalpy ($\:{\varDelta\:\varvec{H}}_{\varvec{a}\varvec{d}\varvec{s}}^{^\circ\:}$) can be calculated as shown in Fig. 7. The negative values of enthalpy of adsorption indicate that the reaction is exothermic^69^. The enthalpy of adsorption is indicated to be in the range of 48.5–58.4 which confirms the formation of both physical and chemical adsorptions^70^. The values of entropy ($\:{\varDelta\:\varvec{S}}_{\varvec{a}\varvec{d}\varvec{s}}^{^\circ\:}$) are decreased up to 313 K, indicating the desorption of water molecules from the surface and exchange with inhibitor molecules. The entropy values confirm the formation of physical and chemical adsorption on steel surfaces^71^. The values of adsorption constant $\:{K}_{ads}$, Gibbs free energy of adsorption $\:\varDelta\:{G}_{ads}^{^\circ\:}$, enthalpy of adsorption $\:\varDelta\:{H}_{ads}^{^\circ\:}$ and entropy of adsorption $\:\varDelta\:{S}_{ads}^{^\circ\:}$ of both inhibitors TAC 6 and TAC 18 were summarized in Table 4.

**Table 4:** Values of thermodynamic adsorption parameters for the inhibitors on steel in 1 M HCl from the mass loss technique at different temperatures.

Surfactant name	Temp., $\:\varvec{K}$	$\:{\varvec{K}}_{\varvec{a}\varvec{d}\varvec{s}}$ L g^− 1^	$\:\varDelta\:{\varvec{G}}_{\varvec{a}\varvec{d}\varvec{s}}^{^\circ\:}$ kJ mol^− 1^	$\:\varDelta\:{\varvec{H}}_{\varvec{a}\varvec{d}\varvec{s}}^{^\circ\:}$ kJ mol^− 1^	*R* ^2^	$\:\varDelta\:{\varvec{S}}_{\varvec{a}\varvec{d}\varvec{s}}^{^\circ\:}$ J mol^− 1^
TAC 6	303	609.8	-26.3	-48.5	0.93	-73.35
	308	400.0	-25.6			-74.26
	313	238.7	-24.7			-76.04
	318	208.8	-24.6			-74.71
	323	191.9	-24.3			-73.05
TAC 18	303	757.6	-26.8	-58.4	0.86	-104.61
	308	289.9	-24.8			-108.97
	313	194.6	-24.2			-109.51
	318	174.8	-24.3			-107.51
	323	164.7	-24.5			-105.21

### Electrochemical techniques

#### Open circuit potential

**Fig. 8:**
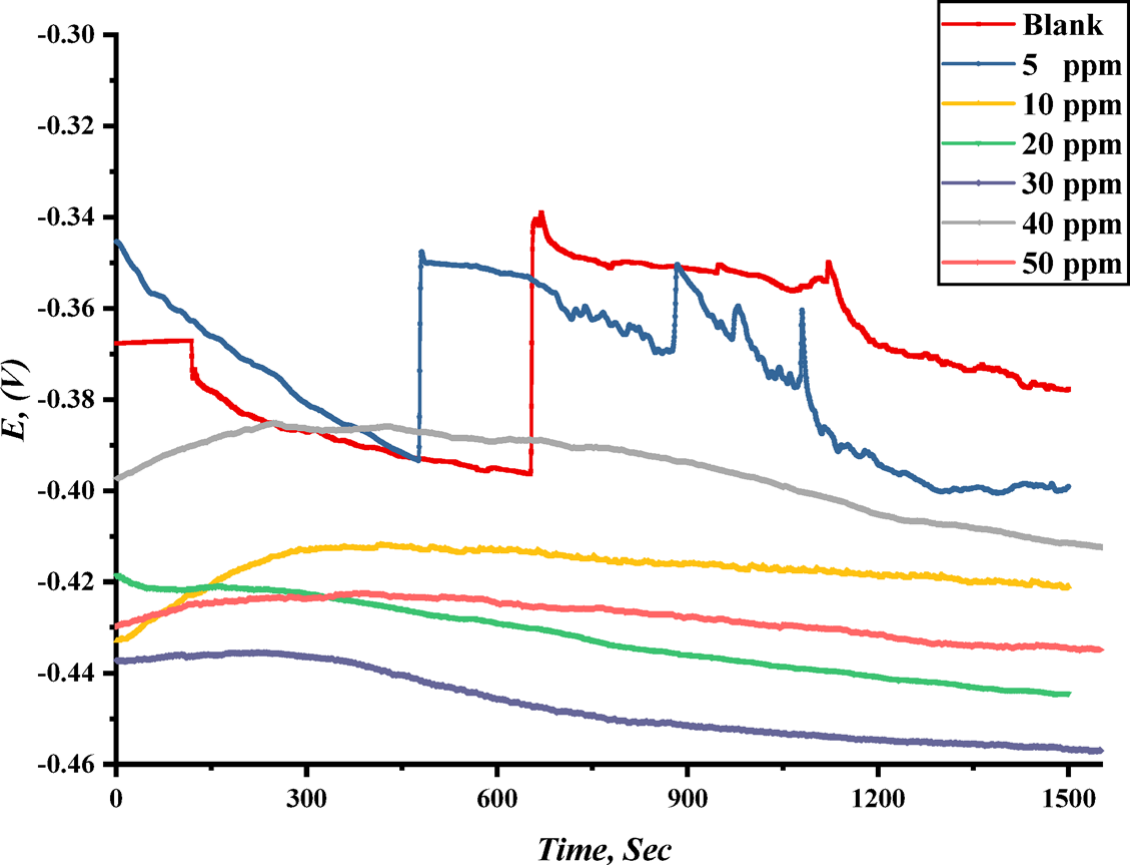
OCP vs. time for TAC cationic Gemini surfactant in 1 M HCl solution at different concentration.

Open Circuit Potential (E_OCP_) measurements provide fundamental insights into the electrochemical processes occurring at the steel-electrolyte interface, serving as a preliminary diagnostic tool for evaluating corrosion behavior and inhibition efficiency for both TAC 6 and TAC 18 inhibitors. Figure 8 illustrates the time-dependent evolution of E_OCP_ for a steel electrode immersed in an aerated 1 M HCl solution, both in the absence and presence of TAC 6 cationic Gemini surfactant at 303 K. In the blank solution, the E_OCP_ stabilizes at a relatively less negative potential (− 0.360 V), which can be attributed to the dissolution of steel in the acidic medium and the subsequent deposition of corrosion products on the metal surface^72^. However, upon adding the TAC 6 inhibitor, the E_OCP_ shifts toward more negative values (from − 0.390 to − 0.450 V), indicating significant adsorption of the TAC 6 inhibitor on the anodic sites of the steel/solution interface^73^. This adsorption hinders metal dissolution, altering the electrochemical dynamics of corrosion. Notably, as the concentration of the extract increases, the E_OCP_ exhibits a progressive shift toward cathodic values, suggesting a dominant cathodic inhibition mechanism^74^. Based on this behavior, TAC 6 and TAC 18 inhibitors can be classified as a cathodic-type corrosion inhibitor^75^.

#### Polarization test

**Fig. 9:**
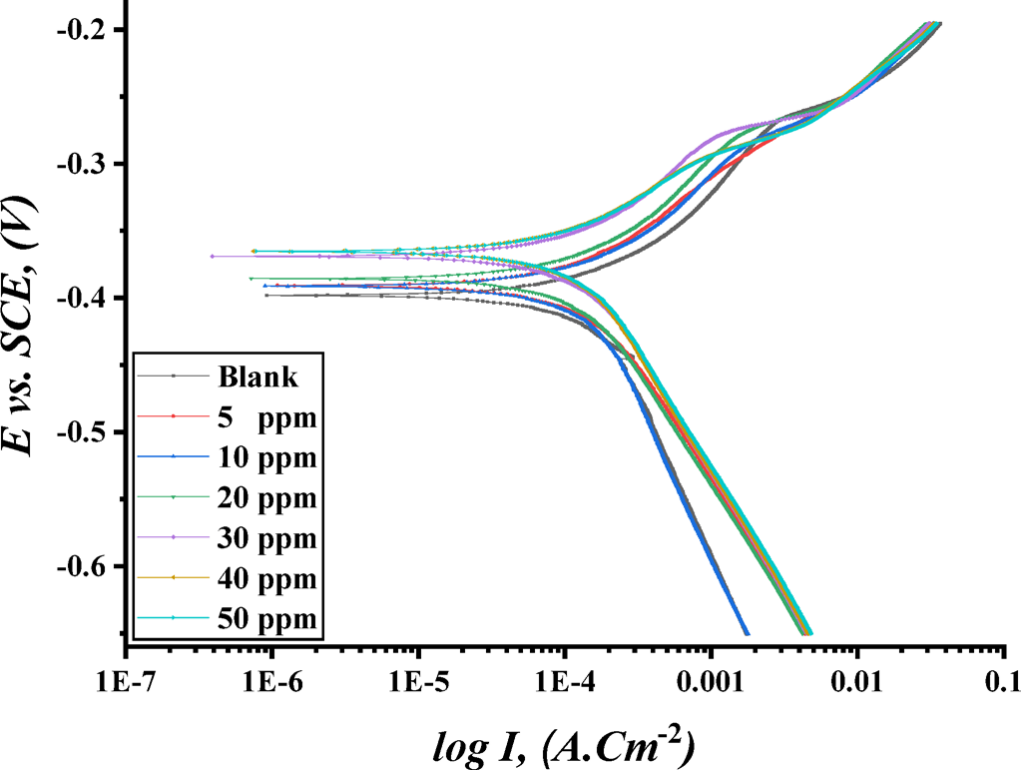
Potentiodynamic polarization curves of carbon steel in 1 M HCl without and with different concentrations of TAC 6 inhibitor.

Tafel polarization curves were used to visualize the corrosion behavior in the steel specimens in a 1 M HCl without and with various concentrations of TAC 6 and TAC 18 as shown in Fig. 9. The parameters of Tafel polarization, including anodic slope ($\:{\beta\:}_{a}$), cathodic slope ($\:{\beta\:}_{c}$), and corrosion current densities ($\:{I}_{corr}$) were detected by extrapolating the curves to the corrosion potential ($\:{E}_{corr}$). The inhibition efficiencies of both inhibitors and other parameters are presented in Table 5. It is noticed that the inhibitors influenced the cathodic reaction more than the anodic reaction^76^. The inhibition efficiencies ($\:\varvec{I}\varvec{E}\:\varvec{\%}$) and surface coverage ($\:\varvec{\theta\:}$) of TAC 6 and TAC 18 are calculated according to Eq. 8.

$\:{\varvec{I}}_{\varvec{i}\varvec{n}\varvec{h}.}$ and $\:{\varvec{I}}_{\varvec{b}}$ are corrosion current densities of inhibited and uninhibited solutions, respectively

8 $$\:IE\:\%=\:\theta\:\times\:100=\left[1-\frac{{I}_{inh.}}{{I}_{b}}\right]\times\:100$$

The displacement of corrosion potential ($\:{E}_{corr}$) can be used to detect the type of inhibitor action. The corrosion potential is larger than 85 mV on more positive or negative sides, indicating that the inhibitors can be anodic or cathodic^77,78^. The displacement of corrosion potential in the presence of both examined inhibitors is indicated to be higher than 85 mV in the cathodic direction^79,80^. This indication revealed that both examined inhibitors are categorized as cathodic inhibitors, affecting the evaluation of hydrogen gas in the cathodic reaction^81,82^. The displacement in the cathodic slope shifts ($\:{\beta\:}_{c}$) is indicated as summarized in Table 5.

**Table 5:** Potentiodynamic polarization parameters for TAC inhibitors in 1 M HCl solution at 303 K.

Surf. Name	Conc. ppm	${I}_{{{corr}\;}} {{\mu A}}~/{{Cm}}^{2}$	${-E}_{{{corr}}}\,{m}{{V}},{{vs}}~{{SCE}}$	$\:{\varvec{\beta\:}}_{\varvec{a}}$ $\:\varvec{m}\mathbf{V}/\varvec{d}\varvec{e}\varvec{c}$	$\:{\varvec{\beta\:}}_{\varvec{c}\:}$ $\:\varvec{m}\mathbf{V}/\varvec{d}\varvec{e}\varvec{c}$	$\:\varvec{I}\varvec{E}\:\varvec{\%}$	$\:\varvec{\theta\:}$
Blank	**0.0**	287.0	389.00	145.0	381.0	**-**	**-**
TAC 6	**5**	182.0	391.00	103.6	191.1	36.59	0.366
	**10**	165.0	391.00	131.0	424.6	42.51	0.425
	**20**	156.0	438.00	103.4	189.0	45.64	0.456
	**30**	151.0	452.00	107.0	140.0	47.39	0.474
	**40**	141.0	365.00	86.00	194.3	50.87	0.509
	**50**	90.00	437.00	112.9	137.4	68.50	0.685
TAC 18	**5**	123.0	445.00	170.6	130.4	57.14	0.571
	**10**	68.0	448.00	145.6	138.0	76.31	0.763
	**20**	57.0	443.00	117.3	119.1	80.14	0.801
	**30**	40.0	447.00	125.6	132.2	86.10	0.861
	**40**	33.0	444.00	105.0	135.0	88.54	0.885
	**50**	35.0	438.00	102.8	132.7	87.80	0.878

#### Electrochemical impedance spectroscopy (EIS)

**Fig. 10:**
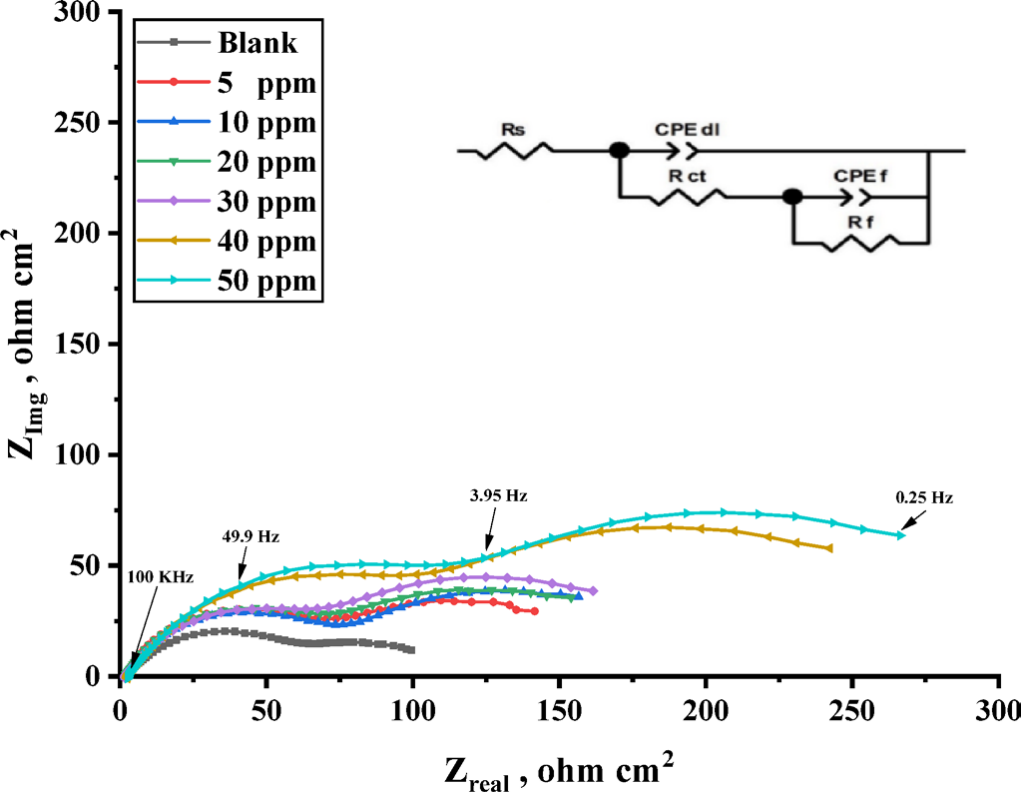
Nyquist plot for carbon steel in 1 M HCl without and with different concentrations of TAC 6 inhibitor.

Figure 10 displays the Nyquist plots of carbon steel immersed in a 1 M HCl solution both before and after the addition of various doses of the investigated inhibitors at 303 K. Due to variations in frequency dispersion or the heterogeneity of the electrochemical system, the impedance in some graphs does not form complete semicircles^83^. The capacitive loop diameter changes with the concentration of the inhibitors; that is, as the concentration increases, the diameter increases^84^. The adsorption of inhibitor molecules on the surface of carbon steel indicates that inhibitor molecules will help establish a stable protective layer^85–87^.

**Fig. 11:**
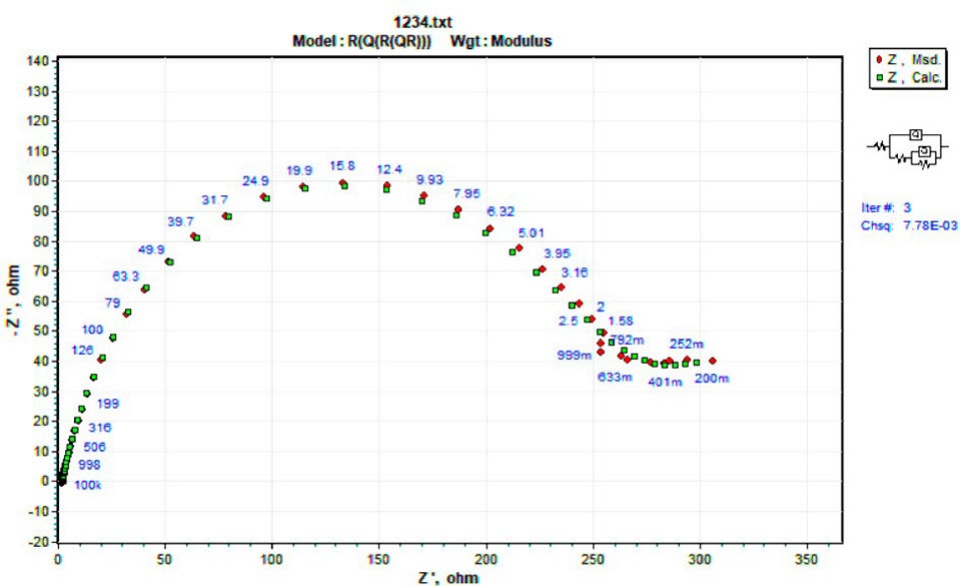
Double CPE equivalent circuit compatible with experimental impedance data.

By analogy, EIS spectra might be understood in terms of electrical impedance. The electrode/electrolyte interface involves distinct processes that may be represented using an analogous electrical circuit, as seen in Fig. 11. This circuit features a constant phase element of double layers (CPE), charge transfer resistance (R_ct_), solution resistance (R_s_), a constant phase element of pores resulting from a defect into the adsorbed layer (C_Po_) and resistance of pores-filled with a conductive corrosive solution (R_Po_). Since the double layer at the interface does not function as a perfect capacitor, the constant phase element is displayed in the circuit in place of the double-layer capacitor to provide a more exact fit^88,89^. Table 6 lists the electrochemical parameters that were ascertained from the impedance plots.

The findings shown in Table 6 demonstrate that when both inhibitors are present, the resistance of charge transfer (R_ct_) values are greater than when the blank solution is used^90,91^. The creation of an insulating protective film at the interface between the solution and carbon steel is attributed to the increase in polarization resistance (R_ct_) values. As the concentration of each inhibitor rises, the value of the constant phase element (CPE) falls, suggesting that there is adsorption on the surface of the carbon steel^92^. Table 6 presents the values of inhibition efficiencies (IE %) and surface coverage ($\:\varvec{\theta\:}$), which were calculated using Eq. 9. Where $R_{{ct}}^{^\circ }$ and $R_{ct}$ are the resistance of charge transfer of uninhibited and inhibited solutions.

9 $$IE~\% = ~\theta \times 100 = \left[ {1 - \frac{{R_{{ct}}^{^\circ } }}{{R_{{ct}} }}} \right] \times 100$$

Through time water molecules and other chloride ions from the aggressive medium can penetrate the film that was adsorbed on a steel surface and formed pores. These pores are filled with the conductive aggressive solution that can produce resistance equal to (R_po_) which indicates to slight increase. The values of R_ct_ > R_po_ for both inhibitors indicate a lesser amount of water molecules substitute inhibitor molecules on the steel surface^93^.

**Fig. 12:**
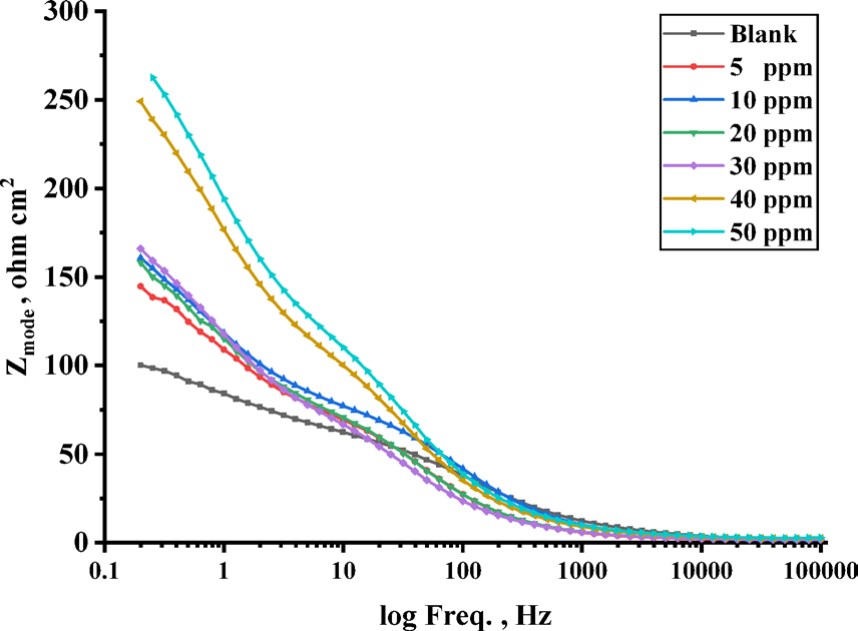
Bode plot for carbon steel in 1 M HCl without and with different concentrations of TAC 6 inhibitor.

Figure 12 displays the carbon steel Bode impedance curves in 1 M HCl both without and with inhibitors at 303 K. The equivalent circuit that was utilized to modify the impedance spectra is established by the appearance of a single peak in the Bode diagrams for both inhibitors and the blank solution^94^. This demonstrates an improvement in the inhibition performance due to the thiazole inhibitors’ measured adsorption on the surface of carbon steel^95^. It is not surprising that the electrochemical experiments and the mass loss approach disagree. It may be understood that in contrast to electrochemical measures, which are reliant on the operating potential, mass loss measurements estimate the corrosion rate irrespective of the electrode potential^96,97^.

**Table 6:** EIS parameter values of C-steel corrosion in 1 M HCl without and with different concentrations at 303 K.

	Conc. (ppm)	$R_{s} \;\Omega .{\text{cm}}^{{\text{2}}}$	$R_{ct} \;\Omega {\text{cm}}^{{\text{2}}}$	$\:CPE$ µF cm^− 2^	$n_1$	$\:{C}_{c}$ µF cm^− 2^	$R_{po} \;\Omega .{\text{cm}}^{{\text{2}}}$	$n_2$	IE %	θ
Blank	0	1.73	83	8071	0.860	10,700	-	1.297	-	-
TAC 6	5	1.73	118	6330	0.142	200.0	6.97E-04	0.829	30.51	0.305
	10	1.75	129	6170	0.149	144.0	3.88E-06	0.831	36.43	0.364
	20	1.79	149	6240	0.153	136.0	5.39E-06	0.815	44.97	0.450
	30	1.64	258	5670	0.178	64.00	1.01E-06	0.779	68.18	0.682
	40	2.14	335	2380	0.110	59.00	4.55E-07	0.869	75.52	0.755
	50	1.87	464	1960	0.103	56.00	9.30E-08	0.841	82.32	0.823
TAC 18	5	1.68	270	1770	0.146	132.0	1.39E-09	0.803	69.63	0.696
	10	1.94	461	1300	0.107	121.0	2.12E-10	0.855	82.21	0.822
	20	2.11	491	619.0	0.034	66.00	6.13E-10	0.801	83.30	0.833
	30	1.80	500	92.00	0.615	22.00	1.30E-10	0.935	83.60	0.836
	40	1.91	618	76.00	0.697	8.000	8.12E-11	0.981	86.73	0.867
	50	1.70	782	66.00	0.066	5.000	1.38E-12	0.005	89.51	0.895

### Surface examination

#### SEM analysis

**Fig. 13:**
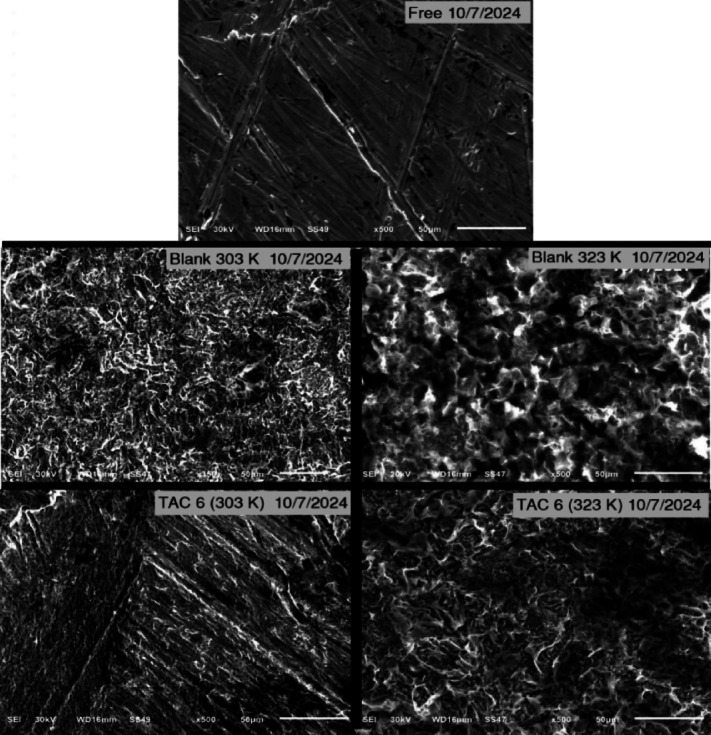
SEM analysis of carbon steel surface before and after immersion in HCl solution without and with the addition of 50 ppm of TAC 6 inhibitor.

The surface morphology of carbon steel specimens was investigated using scanning electron micrographs before and after dipping in HCl for 24 h without and with TAC 6 at two different temperatures (303 and 323 K). Figure 13 depicts the SEM images. The carbon steel surface that was not exposed to a 1 M HCl had a smoother surface than the surface that had been submerged in a 1 M HCl solution. The 1 M acid solution aggressively attacked the exposed surface at 303 K and 323 K, causing significant damage to the steel surface and a multitude of black blotches to cover it. Also, the damage increases as the temperature rises. In contrast, at 303 K and 323 K with the addition of TAC 6 to 1 M HCl, a smoother surface is observed; this is because the inhibitor in the solution forms a shielding film on the surface, slowing down the rate of corrosion. However, carbon steel’s smoothness seemed to diminish as the temperature rose^98^.

#### EDX analysis

**Fig. 14:**
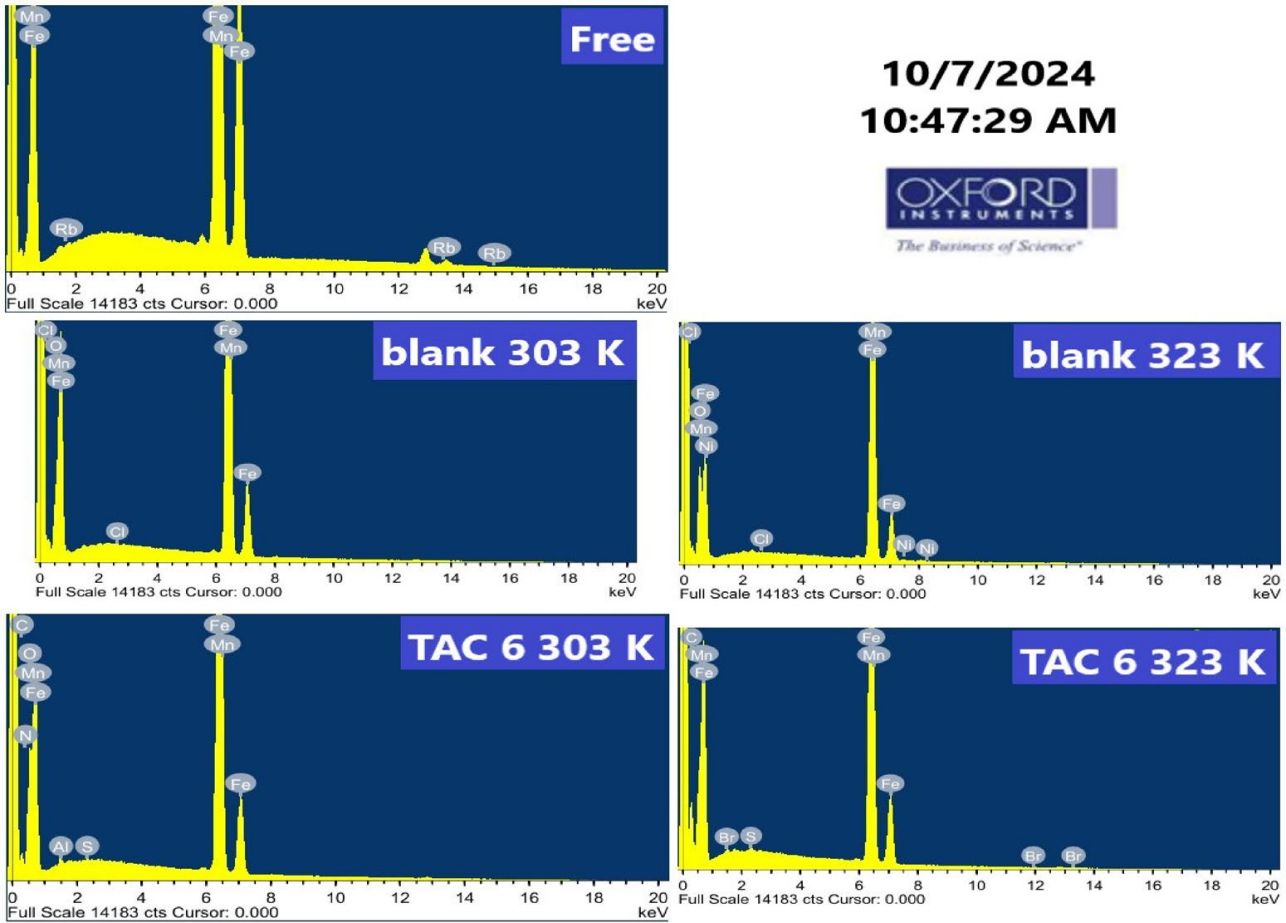
EDX analysis of carbon steel surface before and after immersion in HCl solution without and with the addition of 50 ppm of TAC 6 inhibitor.

The carbon steel specimen surface was examined utilizing energy dispersive X-rays before and after sinking in HCl for 24 h without and with TAC 6 at 303 and 323 K. Figure 14 depicts the EDX analysis. As summarized in Table 7. The weight% of iron in steel specimens decreased more in blank solution at 323 K compared to 303 K. TAC 6 inhibitor was indicated to be adsorbed on the surface with the increase of the weight% of nitrogen, carbon, and sulfur present in the hydrophilic functional group in the Gemini cationic surfactant. The chloride ions indicated to appear again on the steel surface as the temperature increases to 323 K, which can be related to a decrease in adsorption of TAC 6 on the surface. The weight% of nitrogen and oxygen disappears on the steel surface while the iron percentage increases, which refers to the appearance of an uncovered steel surface^99^.

**Table 7:** Weight% of different elements indicated on the surface of steel.

Name of sample	Weight% %							
	Cl	Mn	Fe	O	C	S	Br	*N*
Free	-	0.38	98.80	-	-	-	-	-
Blank − 303 K	0.02	0.46	92.11	7.42	-	-	-	-
Blank − 323 K	0.06	0.44	82.05	16.98	-	-	-	-
TAC 6–303 K	-	0.17	53.13	16.65	17.89	0.03	0.18	11.94
TAC 6–323 K	0.02	0.29	69.84	-	39.50	0.13	0.34	-

#### FTIR analysis

**Fig. 15:**
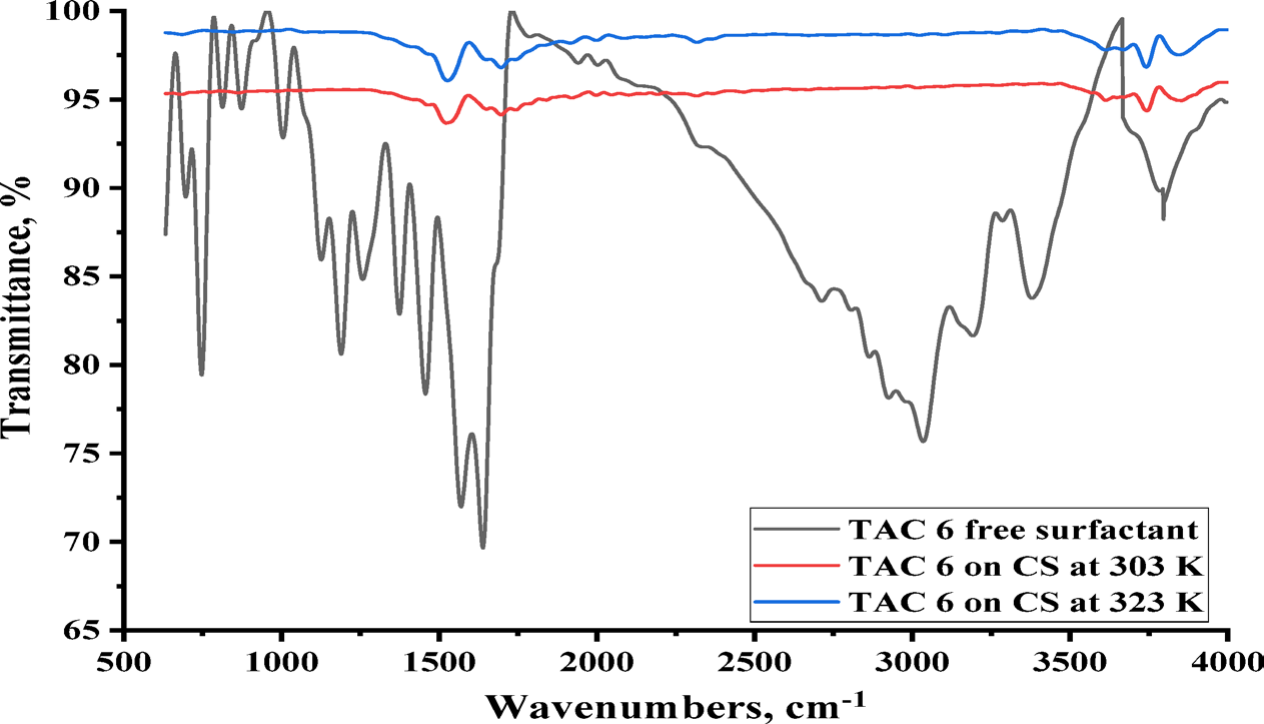
FTIR spectra for TAC 6 inhibitor at 303 K and 323 K.

FT-IR spectra accurately depict the efficient groups of TAC 6 adsorbed on the steel surface. The FTIR findings may be interpreted as shown in Table 8; Fig. 15, which show the layer that develops on carbon steel specimens and the pure solid inhibitor after the samples are soaked in 1 M HCl solution for six hours at two temperatures (303 and 323 K) with 50 ppm of TAC 6 inhibitor present. Following immersion, the spectra of the surface in the presence of the TAC 6 inhibitor are compared to those of the solid inhibitor.

All bands support the theory of forming a monolayer of TAC 6 Gemini cationic surfactant on the steel surface. The band **N**^**+**^**-CH =** indicates the hydrophilic interaction between head groups and steel surfaces. Other compositions of the thiazole ring confirmed the hydrophilic interaction, including -**N-CH=**,** -CH = N-**,** and = C-CH** of the benzene ring or thiazole ring and steel surface. The indication of the **–CH**_**2**_ band confirmed the interaction between an aliphatic chain of TAC 6 surfactant. This indicates the hydrophobic attraction between the inhibitors’ hydrocarbon chains and the steel surface’s increased surface coverage. The transmittance of all adsorbed bands was indicated to decrease with increasing temperature. This decrease supports the decrease in adsorption of TAC 6 Gemini cationic surfactants on the steel surface at 323 K than at 303 K^100^.

**Table 8:** FTIR analysis of adsorbed layer of TAC 6 inhibitor on a steel surface at different temperatures 303 and 323 K.

Vibration	Wavenumbers of TAC 6 (cm^− 1^)		
	Solid surfactant	Adsorbed on CS at 303 K	Adsorbed on CS at 323 K
-CH in a benzene ring	2858	-	-
-CH in methyl group or aliphatic chain	2924	-	-
S-CH = C	3194	-	-
-CH_2_ in the aliphatic chain	3041	-	-
CH_2_-CH_2_ or N-CH=	1642	1693	1698
Benzene -CH = N or CH = CH	1555	1531	1531
=C-CH between the benzene ring and thiazole ring	1424	1466	1457
N^+^-CH = in thiazole ring	1372	1308	1320
CH_2_ in the aliphatic chain	744	685	693

### Quantum chemical calculation

The inhibitors under investigation underwent density-functional theory (DFT) quantum chemical analysis to assess each inhibitor’s effectiveness^101^. Indeed, an inhibitor’s chemical structure determines how effective it is. The inhibitor compounds’ adsorption sites, where they interact with Fe atoms, are predicted by frontier orbital theory. According to reports, the most effective corrosion inhibitors give electrons to vacant orbitals in steel. The adsorption step in the chemical reaction is driven by the contact of the inhibitor’s HOMO and LUMO with the steel surface^102^. Therefore, to understand the inhibitory mechanism, it is crucial to evaluate the existence of HOMO and LUMO orbitals of the compounds under investigation. E_LUMO_ represents a molecule’s ability to accept an electron, whereas E_HOMO_ represents a molecule’s ability to donate electrons.

**Fig. 16:**
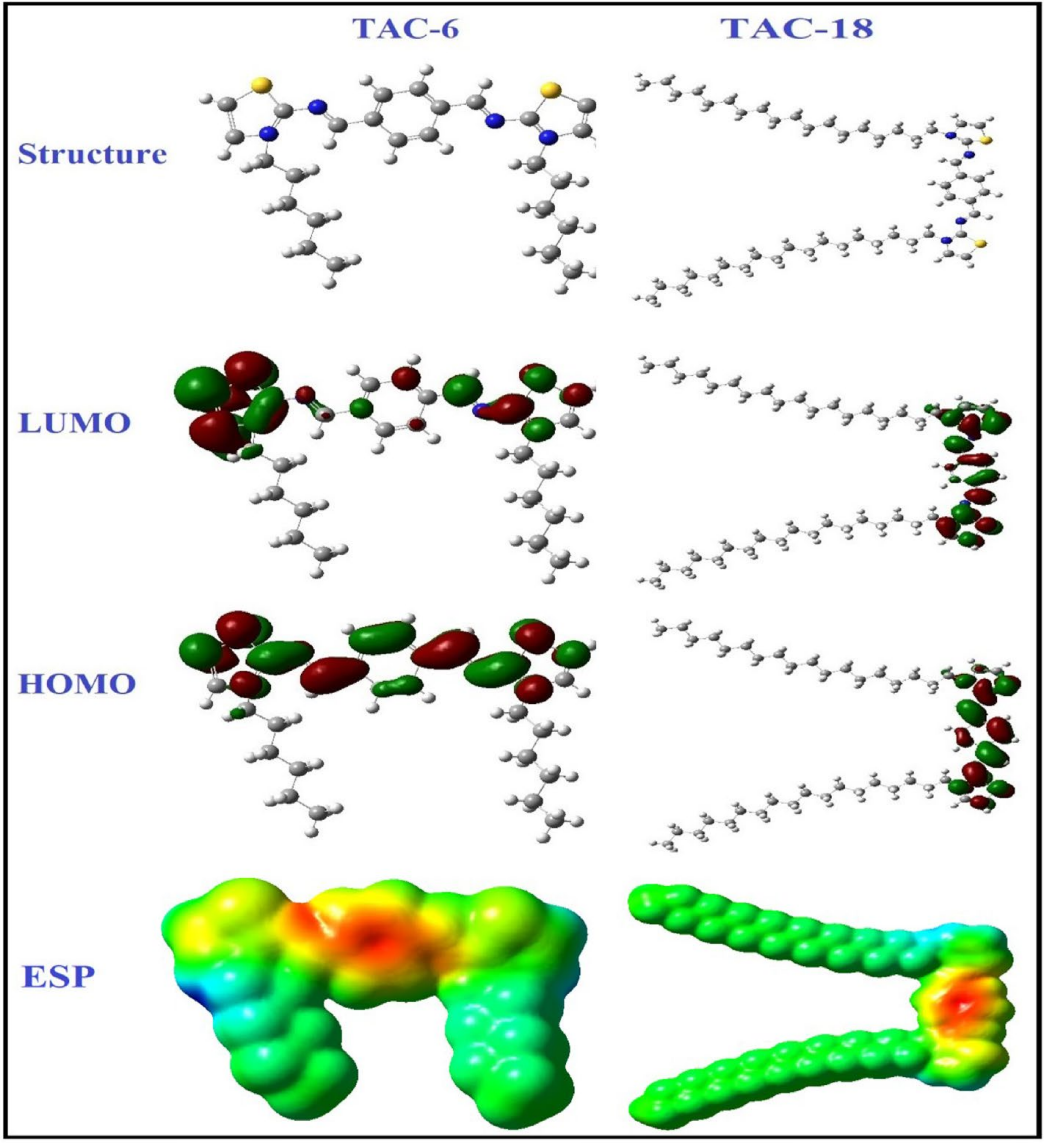
Optimized structure HOMO, LUMO, and ESP of ligand TAC series inhibitors given by the B3LYP/6–31G (d, p) method.

Figure 16 depicts the inhibitors’ geometrical structures, which include multiple HOMO and LUMO locations. Lower ΔE values result in higher inhibition efficiency due to the low energy required to separate electrons from the highest occupied molecular orbital (HOMO). Table 9 demonstrates that low values of ΔE were found for both inhibitors, increasing in the following order: TAC 18 > TAC 6. The quantum chemical calculations were consistent with the experimental results in this configuration, which may be attributed to inhibitor adsorption occurring through a mix of physical and chemical adsorption.

The geometries of the inhibitors TAC 6 and TAC 18 with different HOMO and LUMO values are shown in Fig. 16. This study has demonstrated that better inhibition efficiencies are associated with lower values of ΔE since this indicates a reduced energy need to remove an electron from the highest occupied molecular orbital (HOMO)^103^. Table 9 demonstrates that the three inhibitors had modest values of ΔE, which rose in the following order: TAC 18 > TAC 6. The experimental results in this configuration matched the results of quantum chemical simulations; this confirms that the inhibitors are adsorbed by a mix of chemical and physical adsorption processes^104^. This is because aromatic rings and lone pairs of heteroatoms including sulfur and nitrogen, have greater electron densities.

Furthermore, softness (σ) is a vital factor that can reveal the adsorption capability of the molecules. A higher softness value and a lower hardness value are associated with good contact with the metal and excellent inhibitory effectiveness^105^.

The softness values for both inhibitors indicated the good affinity of the inhibitor molecules for adsorption, which is consistent with the investigational methods. The positive values of energy gaps indicate that the molecules possess a stronger tendency to give electrons to the vacant orbitals in steel^106^. The electrophilic and nucleophilic portions of the molecule are defined by the ESP, which is produced by electron density on the surface. According to distinct colors, the ESP of the molecules TAC inhibitors is determined, as shown in Fig. 16. The blue zone symbolizes a partial positive charge and a strong attraction; the light blue region displays electron deficiency, the yellow region specifies that this area is slightly electron-rich, and the green region is neutral. The red color characterizes the negative potential and designates a strong repulsion. The two thiazole rings have a large concentration of negative charge, making them prime targets for nucleophilic assault. This demonstrates that TAC 6 and TAC18 are primarily resistant to electrophilic attack and have a high ability to adhere to the surface. The calculated quantum parameters are shown in Table 9.

The concept of atomic charge is a useful tool for understanding molecular properties. One of the most widely used computational methods for determining atomic charges is the Mulliken population analysis, which distributes the total electronic charge across the atoms within a molecule^107^. The Mulliken charge distribution for the synthesized compounds is shown in Table 10. Notably, heteroatoms such as oxygen (O), nitrogen (N), and sulfur (S) exhibit the highest charge densities. These regions of elevated electron density are typically the primary sites for electrophilic attack, making O, N, and S the most reactive centers in these molecules. Based on this analysis, it can be inferred that the compounds, in addition to existing in a cationic form that allows electrostatic interaction with the carbon steel surface, may also engage with the carbon steel surface through multiple active sites. This interaction facilitates the formation of a protective layer on the mild steel, effectively slowing down further corrosion in HCl solution.

**Table 9:** Calculated parameters for both inhibitors obtained using the DFT method at the B3LYP/6–31G(d, p) basis set.

Parameters	TAC 6	TAC 18
E_HOMO_ (eV)	-2.70	-2.42
E_LUMO_ (eV)	-1.65	-1.43
ΔE (eV)	1.05	0.99
Ionization (*I*) (eV)	2.70	2.42
Affinity (*A*) (eV)	1.65	1.43
Absolute electronegativity (*χ*)	2.18	1.92
Global hardness (*η*)	0.52	0.50
Softness (*σ*)	1.91	2.01
Δ*N*	4.60	5.10

**Table 10:** Mulliken total atomic charges distribution for TAC 6 and TAC 18.

TAC 6		TAC 18	
Atom	Total charge	Atom	Total charge
C(1)	0.098	C(1)	0.102
C(2)	-0.031	C(2)	-0.029
C(3)	-0.042	C(3)	-0.055
C(4)	0.095	C(4)	0.101
C(5)	-0.034	C(5)	-0.029
C(6)	-0.055	C(6)	-0.055
C(7)	0.081	C(7)	0.041
C(8)	0.047	C(8)	0.04
N(9)	-0.351	N(9)	-0.325
N(10)	-0.316	N(10)	-0.324
S(11)	-0.140	S(11)	-0.106
C(12)	0.440	C(12)	0.439
N(13)	-0.323	N(13)	-0.368
C(14)	0.178	C(14)	0.166
C(15)	0.024	C(15)	0.014
C(16)	0.091	C(16)	0.08
C(17)	-0.038	C(17)	-0.042
C(18)	-0.037	C(18)	-0.04
C(19)	-0.030	C(19)	-0.028
C(20)	-0.032	C(20)	-0.031
C(21)	-0.058	C(21)	-0.028
S(22)	-0.101	C(22)	-0.029
C(23)	0.440	C(23)	-0.028
N(24)	-0.350	C(24)	-0.027
C(25)	0.210	C(25)	-0.029
C(26)	0.040	C(26)	-0.025
C(27)	0.069	C(27)	-0.03
C(28)	-0.030	C(28)	-0.025
C(29)	-0.046	C(29)	-0.029
C(30)	-0.026	C(30)	-0.025
C(31)	-0.034	C(31)	-0.025
C(32)	-0.056	C(32)	-0.028
H(33)	0.016	C(33)	-0.058
H(34)	0.034	S(34)	-0.107
H(35)	0.009	C(35)	0.439
H(36)	0.026	N(36)	-0.369
H(37)	0.003	C(37)	0.166
H(38)	0.008	C(38)	0.015
H(39)	0.055	C(39)	0.079
H(40)	0.060	C(40)	-0.038
H(41)	0.053	C(41)	-0.041
H(42)	0.069	C(42)	-0.028
H(43)	0.038	C(43)	-0.033
H(44)	0.045	C(44)	-0.028
H(45)	0.023	C(45)	-0.03
H(46)	0.025	C(46)	-0.028
H(47)	0.023	C(47)	-0.03
H(48)	0.022	C(48)	-0.031
H(49)	0.017	C(49)	-0.027
H(50)	0.020	C(50)	-0.031
H(51)	0.022	C(51)	-0.025
H(52)	0.021	C(52)	-0.029
H(53)	0.018	C(53)	-0.023
H(54)	0.047	C(54)	-0.023
H(55)	0.058	C(55)	-0.029
H(56)	0.064	C(56)	-0.052
H(57)	0.062	H(57)	0.007
H(58)	0.034	H(58)	0.034
H(59)	0.043	H(59)	0.007
H(60)	0.025	H(60)	0.034
H(61)	0.029	H(61)	0.004
H(62)	0.021	H(62)	0.004
H(63)	0.022	H(63)	0.044
H(64)	0.018	H(64)	0.058
H(65)	0.021	H(65)	0.052
H(66)	0.021	H(66)	0.06
H(67)	0.02	H(67)	0.035
H(68)	0.02	H(68)	0.046
Br(69)	-0.445	H(69)	0.025
Br(70)	-0.45	H(70)	0.025
-	-	H(71)	0.02
-	-	H(72)	0.021
-	-	H(73)	0.018
-	-	H(74)	0.018
-	-	H(75)	0.016
-	-	H(76)	0.017
-	-	H(77)	0.016
-	-	H(78)	0.015
-	-	H(79)	0.014
-	-	H(80)	0.015
-	-	H(81)	0.015
-	-	H(82)	0.014
-	-	H(83)	0.014
-	-	H(84)	0.015
-	-	H(85)	0.015
-	-	H(86)	0.014
-	-	H(87)	0.013
-	-	H(88)	0.014
-	-	H(89)	0.015
-	-	H(90)	0.014
-	-	H(91)	0.013
-	-	H(92)	0.014
-	-	H(138)	0.018
-	-	H(139)	0.016
-	-	H(140)	0.014
-	-	Br(141)	-0.379
-	-	Br(142)	-0.318

### Synergistic effect of salts using electrochemical methods

#### Potentiodynamic polarization

**Fig. 17:**
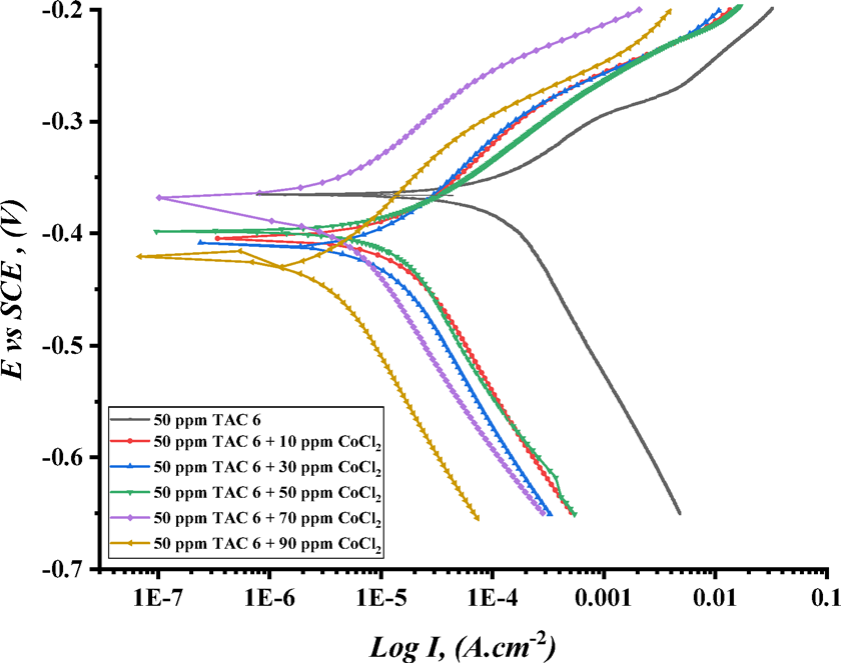
Potentiodynamic polarization curves ($\:\text{log}I\:$ plotted against $\:E\:vs.\:SCE$) Of C-steel in 1 M HCl for TAC 6 surfactant at 50 ppm with the addition of different concentrations of CoCl_2_.

**Fig. 18:**
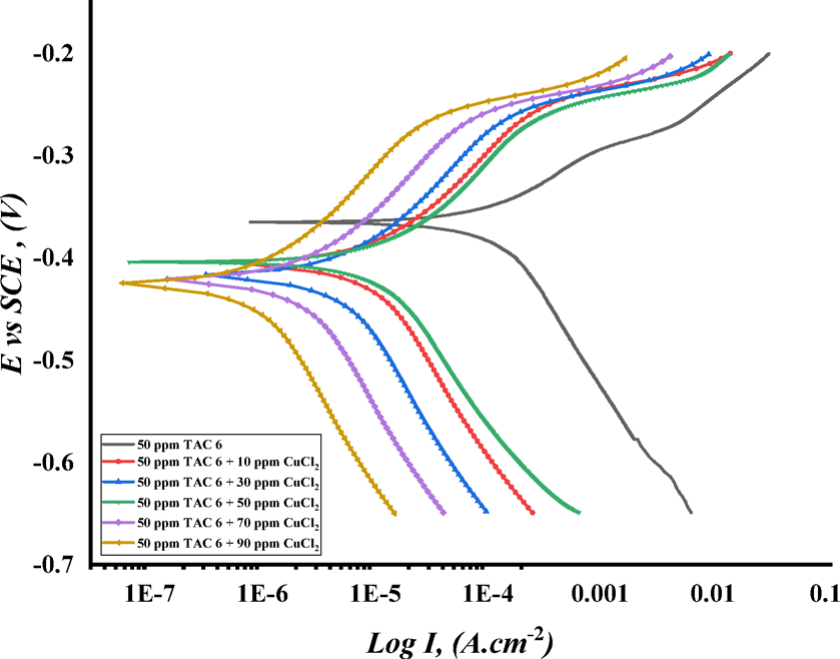
Potentiodynamic polarization curves ($\:\text{log}I\:$ plotted against $\:E\:vs.\:SCE$) Of C-steel in 1 M HCl for TAC 6 surfactant at 50 ppm with the addition of different concentrations of CuCl_2_.

**Fig. 19:**
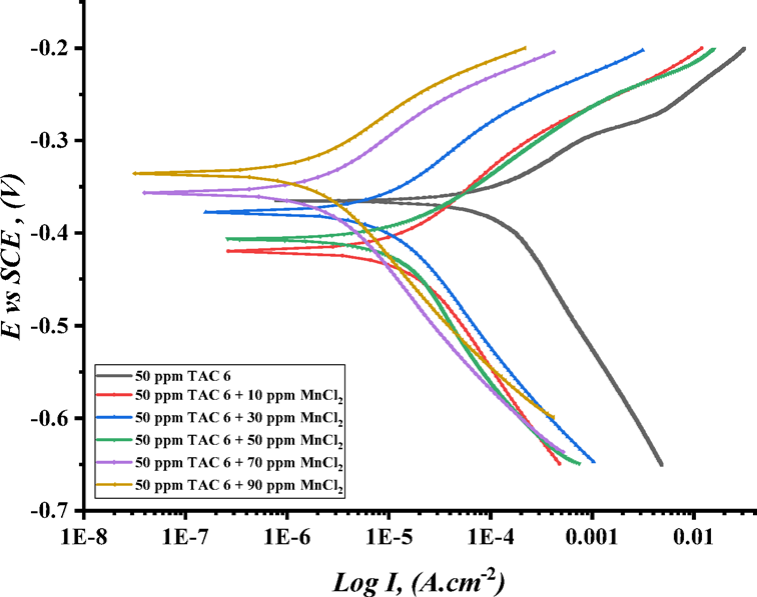
Potentiodynamic polarization curves ($\:\text{log}I\:$ plotted against $\:E\:vs.\:SCE$) of C-steel in 1 M HCl for TAC 6 surfactant at 50 ppm with the addition of different concentrations of MnCl_2_.

Figures 17, 18 and 19 illustrate the polarization curves of steel in HCl in the presence of TAC 6 and TAC 18, as well as the addition of various amounts of salts CoCl_2_, MnCl_2_, and CuCl_2_. The polarization parameters were estimated. Table 11 summarizes the corrosion potential (E_corr_), current density (I_corr_), and cathodic and anodic Tafel slopes (βc, βa).

When seen in Figs. 17, 18 and 19, both anodic and cathodic curves change toward falling current density (I_corr_) when the addition of different salts increases^108^, and the pattern is more noticeable in the following arrangement: CuCl_2_ > MnCl_2_ > CoCl_2_^109,110^. This could be related to how all of the inhibitors adsorb synergistically when salts are present on carbon steel surfaces^111^. Thorough research reveals that, as shown in Table 11, the addition of various salts adds to an increase in the inhibitory efficacy of both inhibitors under consideration.

The results imply that different salts change the mechanism of inhibitors’ works from affecting the cathodic reaction to affecting both anodic and cathodic slopes (β_c_) and (β_a_). Moreover, the moderate change in the corrosion potential (E_corr_) indicated to be mixed-type inhibitors^112^. The synergistic action of salts showed a reduction in steel dissolving and hydrogen evaluation corrosion rate^113^. The salts impacted the rise in shield layers adsorbed on the surface, indicating an increase in surface coverage and inhibition efficiency^114^. Salts can influence Gemini cationic surfactant adsorption on the steel by one of two mechanisms: cooperative adsorption or competitive adsorption^115^. According to the synergistic effect, cooperative adsorption is recommended to raise the inhibitions of all surfactants. Inhibitors are adsorbed on the layer that the chloride ions produce when chloride anions in salts first chemisorb on the steel surface. In competitive adsorption inhibitors, and salts engage in an antagonistic competition for adsorption on steel surfaces, which lowers the inhibitors’ efficiency. The cation components of the Gemini cationic surfactants are adsorbed on a layer of the chloride ions that forms when the chloride ions from various salts, such as CoCl_2_, MnCl_2_, and CuCl_2_, are adsorbed on the surface^116^. The synergistic inhibitory effect is caused by the significant adsorption of bromide ions from inhibitors and chloride ions of various salts, such as CoCl_2_, MnCl_2_, and CuCl_2_. These ions are on the surface of carbon steel. Columbic attraction then adsorbs the cationic portion of the Gemini cationic surfactant onto the carbon steel surface. The whole adsorption of inhibitor molecules, bromide ions, and chloride ions causes the surface covering of carbon steel to rise, which in turn raises the inhibition efficiencies^117^.

**Table 11:** Data from PP test for corrosion of C-Steel in 1 M HCl at 50 Ppm of the inhibitors in the presence of different concentrations of all salt at 303 K.

Salt name	Inhibitors name	Conc. ppm	$I_{{corr}} \;\mu A~Cm^{{ - 2}}$	$- ~E_{{corr}} \;mV$	$\:{\varvec{\beta\:}}_{\varvec{a}}$ $\:\varvec{m}\mathbf{V}/\varvec{d}\varvec{e}\varvec{c}$	$\:{\varvec{\beta\:}}_{\varvec{c}\:}$ $\:\varvec{m}\varvec{V}/\varvec{d}\varvec{e}\varvec{c}$	$\:IE\:\%$	$\:\varvec{\theta\:}$
CoCl_2_	TAC 6	**0**	**90.00**	**437**	**112.9**	**137.4**	**68.50**	**0.685**
		**10**	46.29	405	86.6	170.8	88.10	0.881
		**30**	39.23	409	92.1	179.7	89.92	0.899
		**50**	27.05	400	75.6	151.2	93.05	0.930
		**70**	24.78	369	72.8	167.9	93.63	0.936
		**90**	22.52	420	71.2	165.4	94.21	0.942
	TAC 18	**0**	**35.00**	**438**	**102.8**	**132.7**	**87.80**	**0.878**
		**10**	24.06	423	79.2	150.3	93.81	0.938
		**30**	23.07	445	79.6	157.8	94.07	0.941
		**50**	21.70	469	79.7	165.3	94.42	0.944
		**70**	19.64	510	85.2	180.3	94.95	0.950
		**90**	16.71	382	75.1	175.2	95.70	0.957
MnCl_2_	TAC 6	**0**	**90.00**	**437**	**112.9**	**137.4**	**68.50**	**0.685**
		**10**	25.31	419	70.00	202.1	93.49	0.935
		**30**	23.98	379	60.00	181.9	93.84	0.938
		**50**	21.30	407	60.00	171.8	94.52	0.945
		**70**	20.53	357	50.00	161.7	94.72	0.947
		**90**	19.42	336	60.00	163.7	95.01	0.950
	TAC 18	**0**	**35.00**	**438**	**102.8**	**132.7**	**87.80**	**0.878**
		**10**	15.94	434	74.20	131.0	95.90	0.959
		**30**	15.83	391	66.80	117.9	95.93	0.959
		**50**	13.70	396	63.80	97.70	96.48	0.965
		**70**	11.32	372	63.10	111.4	97.09	0.971
		**90**	9.75	348	59.40	104.8	97.49	0.975
CuCl_2_	TAC 6	**0**	**90.00**	**437**	**112.9**	**137.4**	**68.50**	**0.685**
		**10**	34.04	412	112.2	188.9	91.25	0.913
		**30**	23.82	418	106.6	179.4	93.88	0.939
		**50**	18.41	417	109.7	161.4	95.27	0.953
		**70**	15.52	419	96.50	162.4	96.01	0.96
		**90**	13.56	425	92.00	154.9	96.51	0.965
	TAC 18	**0**	**35.00**	**438**	**102.8**	**132.7**	**87.80**	**0.878**
		**10**	13.52	418	73.30	129.1	96.53	0.965
		**30**	11.66	377	66.00	116.2	97.00	0.970
		**50**	10.54	400	60.10	115.9	97.29	0.973
		**70**	9.620	357	62.30	109.7	97.53	0.975
		**90**	8.310	335	58.70	103.4	97.86	0.979

#### Electrochemical impedance spectroscopy

**Fig. 20:**
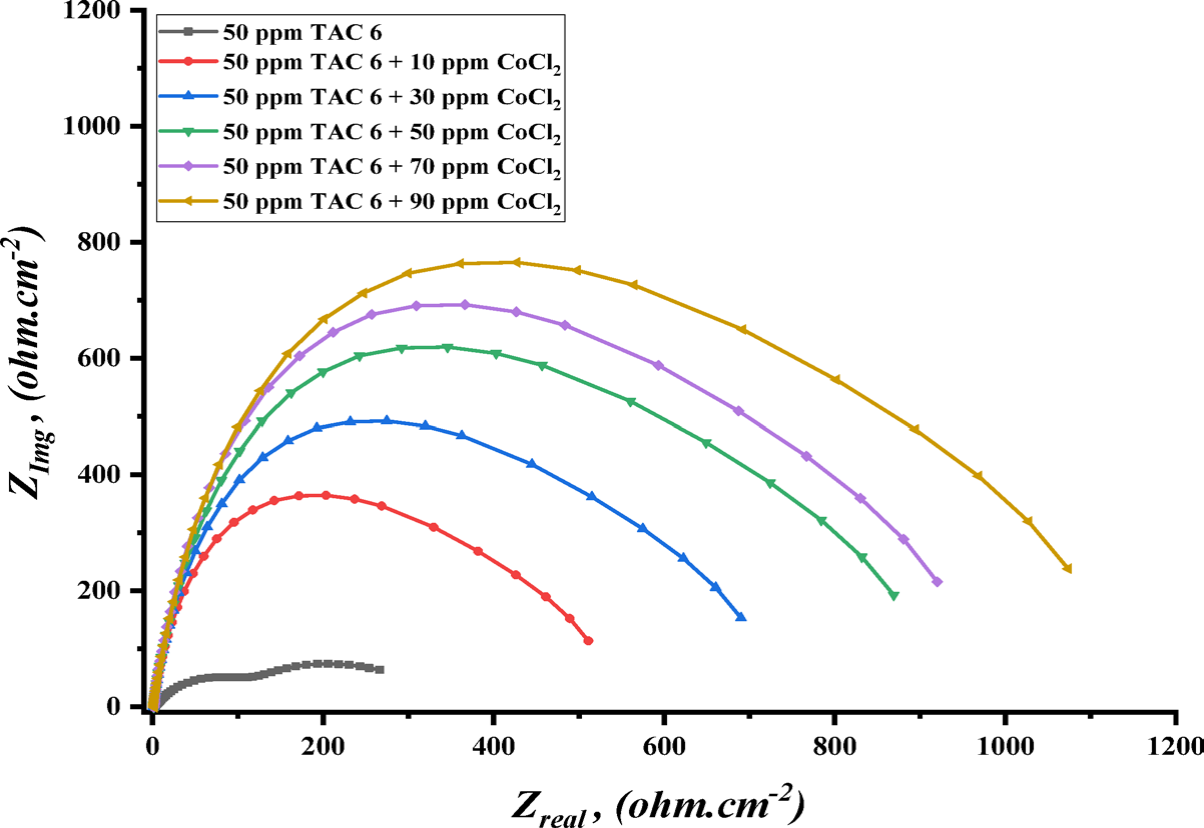
Nyquist plot of carbon steel in 1 M HCl with 50 ppm of TAC 6 inhibitor in the presence of different concentrations of CoCl_2_ salt (10–90 ppm).

**Fig. 21:**
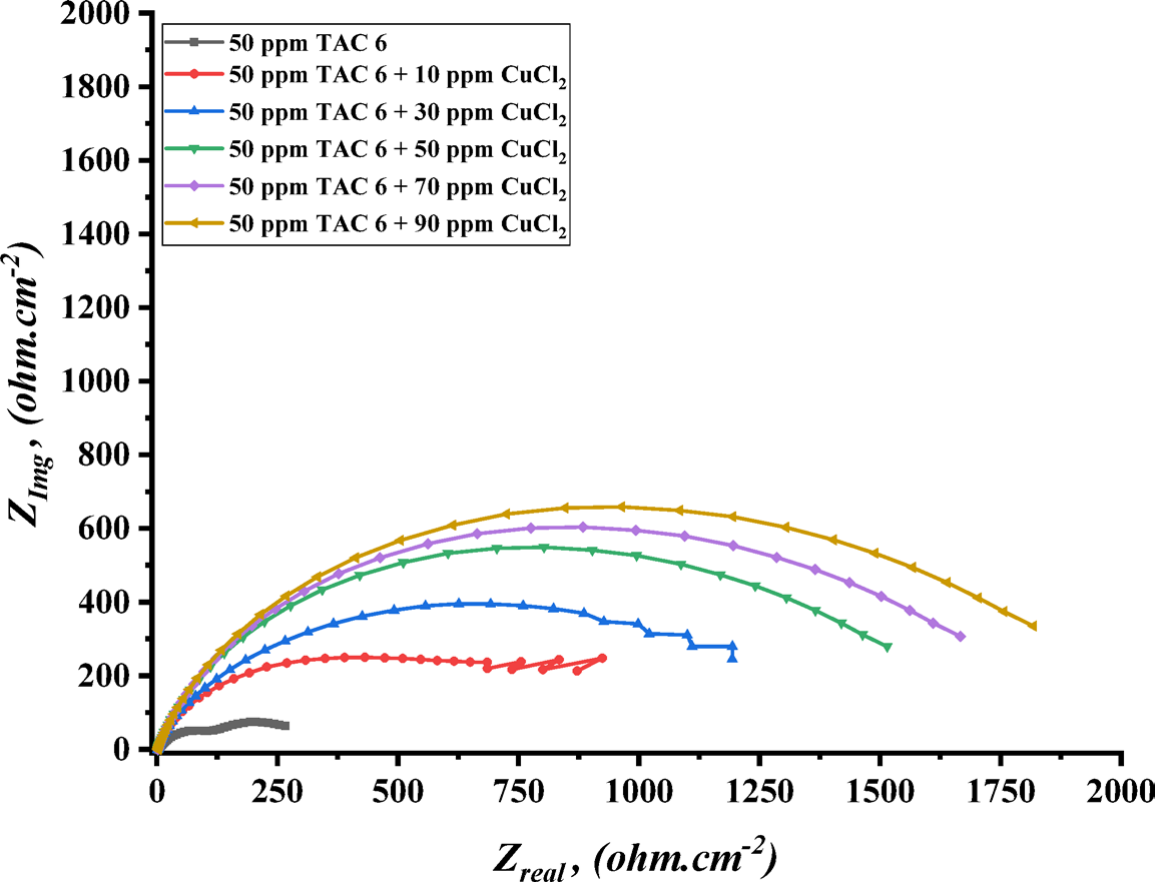
Nyquist plot of carbon steel in 1 M HCl with 50 ppm of TAC 6 inhibitor in the presence of different concentrations of CuCl_2_ salt (10–90 ppm).

**Fig. 22:**
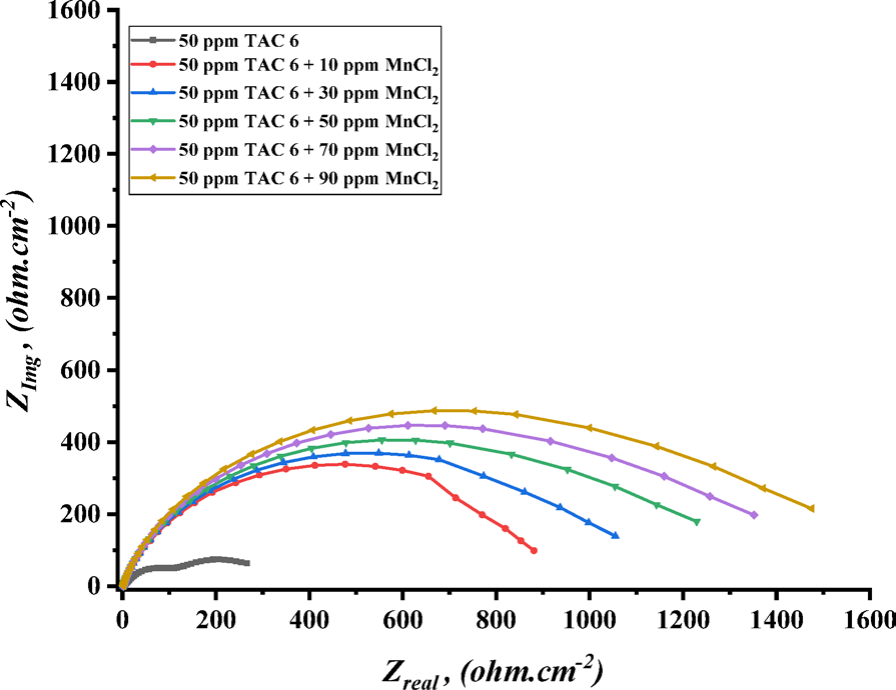
Nyquist plot of carbon steel in 1 M HCl with 50 ppm of TAC 6 inhibitor in the presence of different concentrations of MnCl_2_ salt (10–90 ppm).

**Fig. 23:**
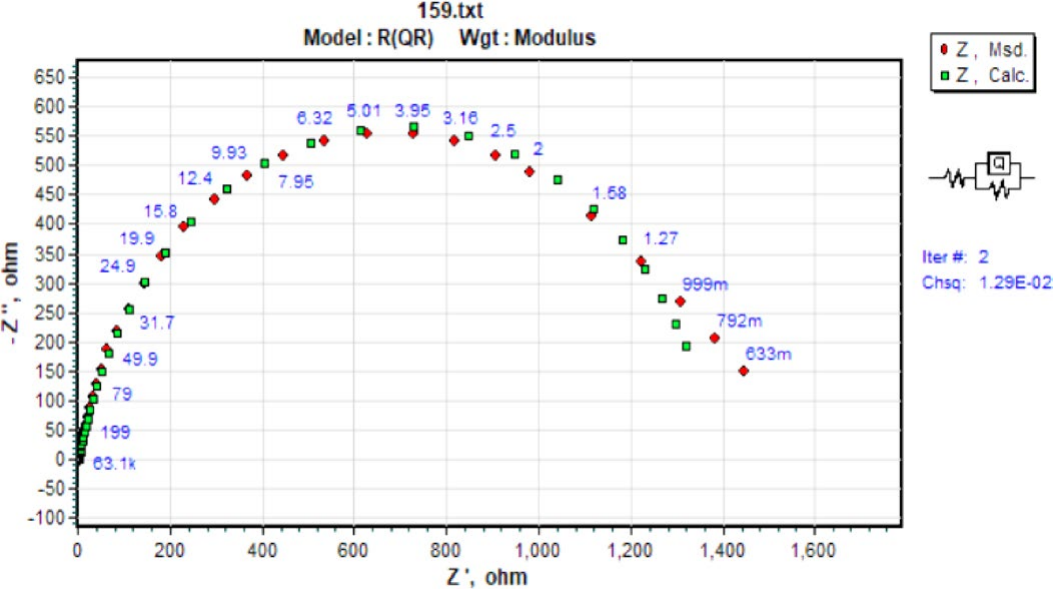
CPE equivalent circuit compatible with the experimental impedance data.

**Fig. 24:**
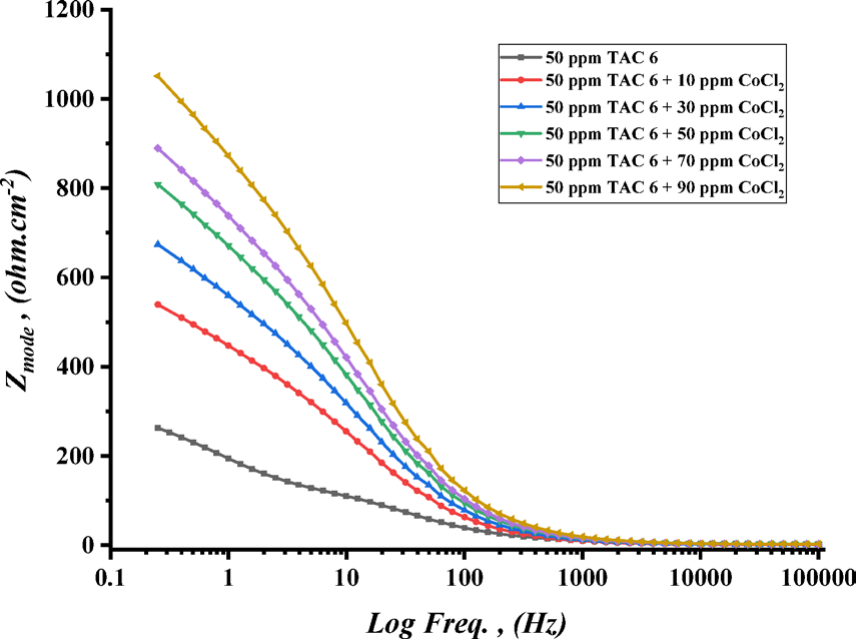
Bode plot of carbon steel in 1 M HCl with 50 ppm of TAC 6 inhibitor in the presence of different concentrations of CoCl_2_ salt (10–90 ppm).

**Fig. 25:**
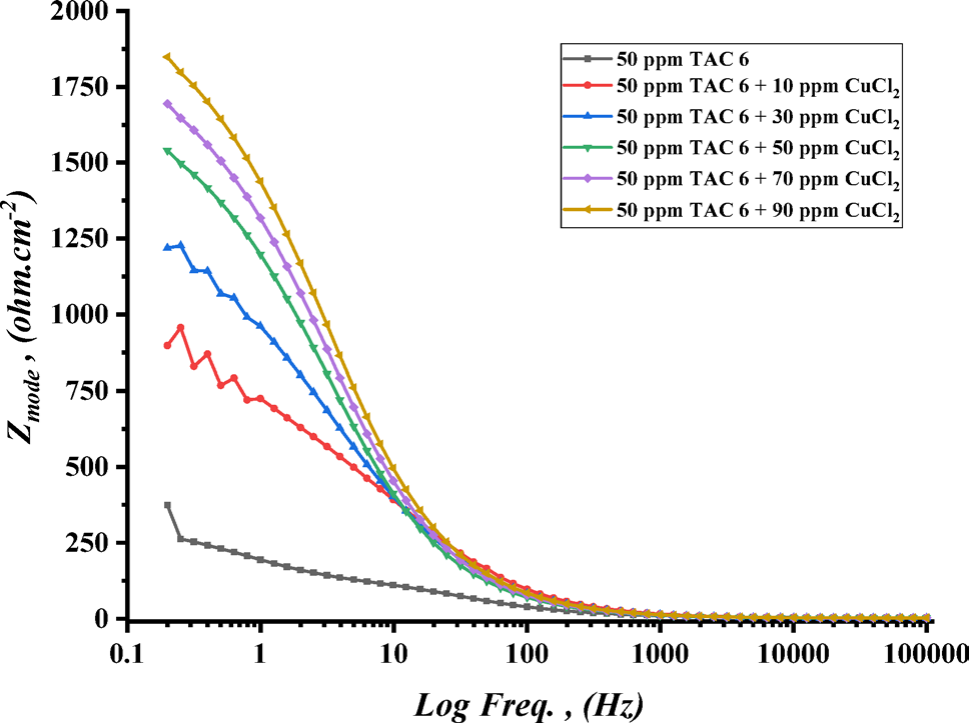
Bode plot of carbon steel in 1 M HCl with 50 ppm of TAC 6 inhibitor in the presence of different concentrations of CuCl_2_ salt (10–90 ppm).

**Fig. 26:**
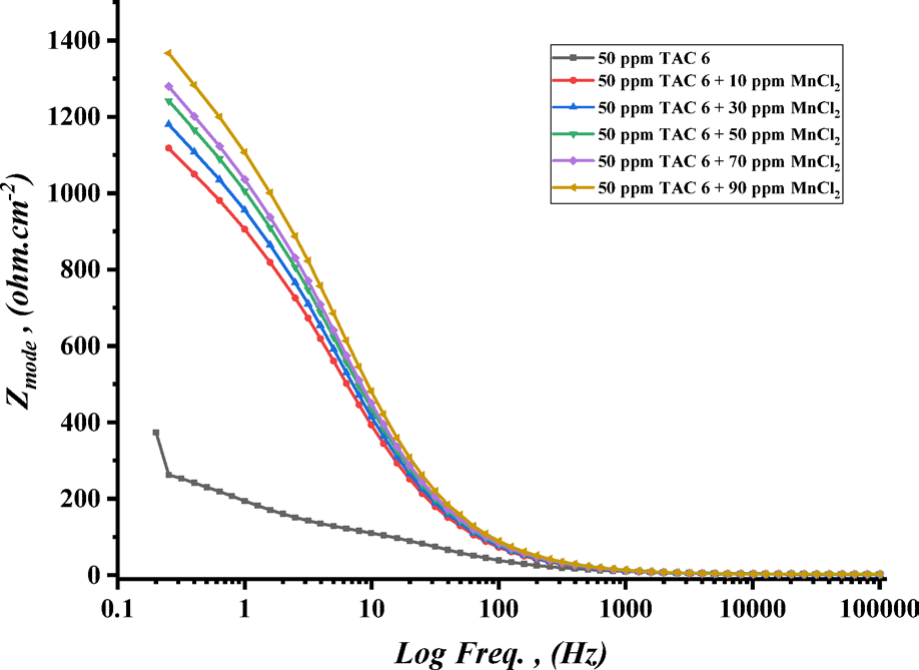
Bode plot of carbon steel in 1 M HCl with 50 ppm of TAC 6 inhibitor in the presence of different concentrations of MnCl_2_ salt (10–90 ppm).

The impedance experiments were achieved to explain the synergic interactions between the inhibitor and different concentrations of salts, including CoCl_2_, MnCl_2_, and CuCl_2_ as summarized in Table 12. The Nyquist plots for steel in the presence of TAC 6 and TAC 18 inhibitors in combination with different salts CoCl_2_, MnCl_2_, and CuCl_2_ are shown in Figs. 20, 21 and 22. The increase of the salt concentrations in combination with the examined inhibitors leads to an increase in the diameter of the semicircle, indicating the inhibition of the corrosion reaction^118^. The impedance data were fitted to the equivalent circuit model mentioned in Fig. 23, which proved more accurate^119^. The polarization resistance was indicated to increase while constant phase electrode CPE was diminished with a rise of the concentrations of the salts. The decrease in CPE can be related to the decrease in the dielectric constant and increasing the double-layer thickness with the adsorption of both salts and inhibitors on the surface of carbon steel^120^. As summarized in Table 12. CPE decreases as a result of halide ions, including chloride from salts and bromide from inhibitors, replacing water molecules adsorbed on steel surfaces. The efficiencies of inhibition are indicated to be as follows: CuCl_2_ < MnCl_2_ < CoCl_2_^121^. This confirms the inhibition increases with increasing the size of ions that lead to more surface coverages of salts on steel surfaces^122^. The bode plots for both inhibitors in the presence of different salts are shown in Figs. 24, 25 and 26.

The inhibition efficiency trend CuCl₂ > MnCl₂ > CoCl₂ observed in the study deviates from the expected, revealing complex electrochemical interactions between metal cations, surfactant inhibitors, and the steel surface. As anticipated, Cu²⁺ exhibited the highest inhibition efficiency due to its strong adsorption affinity, smaller ionic radius (0.73 Å)^123^, and ability to form a stable CuCl₂ protective layer, leading to increased charge transfer resistance (R_ct_) and reduced corrosion current (I_corr_)^124^. However, Mn²⁺ unexpectedly outperformed Co²⁺, likely due to stronger electrostatic interactions with chloride (Cl⁻) and bromide (Br⁻) ions from the Gemini surfactants (**TAC 6 and TAC 18**), which enhanced inhibitor adsorption and surface coverage. Mn²⁺ may have facilitated ionic bridging, allowing for better cooperative adsorption with the inhibitors, resulting in a thicker and more protective layer on the steel surface^125^. In contrast, Co²⁺, despite having a slightly smaller ionic radius than Mn²⁺ (0.79 Å vs. 0.83 Å)^126^, may not have formed as stable surface complexes or contributed as effectively to surfactant adsorption, leading to lower inhibition efficiency.

Cu²⁺ has a higher charge density than Mn²⁺ and Co²⁺, allowing it to interact more effectively with electron-rich functional groups in the inhibitors (e.g., nitrogen and sulfur atoms in the thiazole ring)^127^. This leads to stronger complexation, enhancing the stability of the inhibitor-metal interaction^128^. Additionally, Mn²⁺ unexpectedly exhibited higher R_ct_ and lower I_corr_ than Co²⁺, further suggesting its superior adsorption behavior. The overall trend indicates that while Cu²⁺ remains the most effective due to its strong surface affinity and protective complex formation, Mn²⁺ surpassed Co²⁺ due to its enhanced synergistic interactions with the inhibitor molecules and the steel surface, highlighting the importance of electrochemical adsorption mechanisms beyond simple charge density and ionic size considerations.

**Table 12:** EIS parameter values of C-steel corrosion in 1 M HCl at 50 Ppm of the inhibitors with the addition of different concentrations of salts at 303 K.

Salt name	Inhibitors name	Conc. of salt ppm	$R_{p} \;\Omega {\text{cm}}^{{\text{2}}}$	*CPE* F cm^− 2^	$\:\varvec{I}\varvec{E}\:\varvec{\%}$	θ
CoCl_2_	TAC 6	0	464	0.0000663	73.66	0.737
		10	539	0.0000241	84.79	0.848
		30	774	0.0000193	89.41	0.894
		50	909	0.0000161	90.98	0.910
		70	990	0.0000146	91.72	0.917
		90	1200	0.0000124	93.17	0.932
	TAC 18	0	782	0.0000261	90.70	0.907
		10	1050	0.0000200	92.19	0.922
		30	1100	0.0000170	92.55	0.925
		50	1180	0.0000142	93.05	0.931
		70	1450	0.0000024	94.34	0.943
		90	1600	0.0000014	94.88	0.949
MnCl_2_	TAC 6	0	464	0.0000663	73.66	0.737
		10	1018	0.0000030	91.94	0.919
		30	1180	0.0000024	93.05	0.931
		50	1242	0.0000021	93.40	0.934
		70	1391	0.0000020	94.10	0.941
		90	1499	0.0000019	94.53	0.945
	TAC 18	0	782	0.0000261	90.70	0.907
		10	1281	0.0000034	93.60	0.936
		30	1344	0.0000030	93.90	0.939
		50	1407	0.0000026	94.17	0.942
		70	1548	0.0000024	94.70	0.947
		90	1618	0.0000023	94.93	0.949
CuCl_2_	TAC 6	0	464	0.0000663	73.66	0.737
		10	898	0.0000120	90.87	0.909
		30	1219	0.0000067	93.27	0.933
		50	1540	0.0000033	94.68	0.947
		70	1794	0.0000030	95.43	0.954
		90	1948	0.0000026	95.79	0.958
	TAC 18	0	782	0.0000261	90.70	0.907
		10	1502	0.0000035	94.54	0.945
		30	1567	0.0000024	94.77	0.948
		50	1731	0.0000033	95.26	0.953
		70	1894	0.0000017	95.67	0.957
		90	1957	0.0000023	95.81	0.958

### Mechanism of corrosion inhibitors

**Fig. 27:**
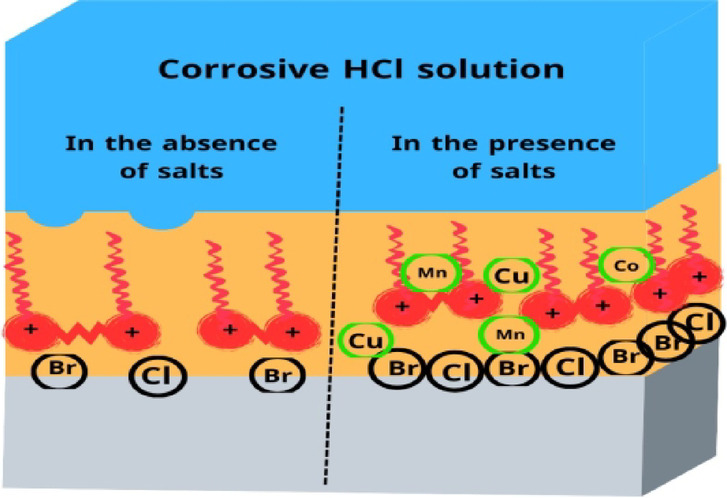
The process by which a steel surface is protected against corrosion in a 1 M HCl solution.

The inhibition inertia of Gemini cationic surfactants is created by forming a protective layer that is absorbed on the steel surface, which obeys the Langmuir adsorption isotherm as shown in Fig. 27. By utilizing the π-electrons in the benzene ring and the vacant d-orbital of the iron atoms, as well as the donor-acceptor interactions between the atoms on the steel surface and the lone electron pairs in heteroatoms like nitrogen and sulfur, the inhibitor molecules can interact with the surface of the steel.

The adsorption on steel surfaces includes mainly electrostatic interactions with the addition of the formation of a chemical bond. All measurements, including chemical, electrochemical, surface examination, and computational, confirmed the mechanism of steel corrosion in 1 M HCl solution reduced with the addition of various concentrations of both Gemini cationic surfactants. Impedance measurement indicates the formation of a film on the steel surface with the creation of small defects in these layers over time. An increase in the inhibition efficiencies is indicated as follows $\:\text{T}\text{A}\text{C}\:18\:>\text{T}\text{A}\text{C}\:6$. The addition of inorganic salts changes the mechanism of inhibitors’ work to act as mixed-type inhibitors. Where cooperative adsorption of chloride ions from examined salts and bromide ions from inhibitors on the steel surface, then the cationic portion of inhibitors is attracted to them while cations of salts cover the surface of steel according to their sizes to form a more uniform protective film on steel surface as follows CuCl₂ > MnCl₂ > CoCl₂.

The mechanism of corrosion of carbon steel in hydrochloric acid solution is through the adsorption of chloride ions, hydrogen ions, and water molecules on the steel surface. The adsorption molecules lead to steel dissolution as an anodic reaction and hydrogen evaluation through cathodic reaction^129^.

**Water adsorption on a steel surface**

$$\:\text{F}\text{e}+{\text{H}}_{2}\text{O}\iff\:{\left[\text{F}\text{e}\text{O}\text{H}\right]}_{\text{a}\text{d}\text{s}}+{\text{H}}^{+}+{\text{e}}^{-}$$

$$\:{\left[\text{F}\text{e}\text{O}\text{H}\right]}_{\text{a}\text{d}\text{s}}\iff\:{{\left[\text{F}\text{e}\text{O}\text{H}\right]}_{\text{a}\text{d}\text{s}}}^{+}+{\text{e}}^{-}$$

$$\:{{\left[\text{F}\text{e}\text{O}\text{H}\right]}_{\text{a}\text{d}\text{s}}}^{+}+{\text{H}}^{+}\iff\:{\text{F}\text{e}}^{2+}+{\text{H}}_{2}\text{O}$$

**Chloride ions adsorption on steel surface**

$$\:\text{F}\text{e}+{\text{H}}_{2}\text{O}+{\text{C}\text{l}}^{-}\iff\:{\left[\text{F}\text{e}\text{C}\text{l}\text{O}\text{H}\right]}_{\text{a}\text{d}\text{s}}^{-}+{\text{H}}^{+}+{\text{e}}^{-}$$

$$\:{\left[\text{F}\text{e}\text{C}\text{l}\text{O}\text{H}\right]}_{\text{a}\text{d}\text{s}}^{-}\iff\:{\left[\text{F}\text{e}\text{C}\text{l}\text{O}\text{H}\right]}_{\text{a}\text{d}\text{s}}+{\text{e}}^{-}$$

$$\:\left[\text{F}\text{e}\text{C}\text{l}\text{O}\text{H}\right]+{\text{H}}^{+}\iff\:{\text{F}\text{e}}^{2+}+{\text{C}\text{l}}^{-}+{\text{H}}_{2}\text{O}$$

**Hydrogen evaluation**

$$\:\text{F}\text{e}+\:{\text{H}}^{+}\:\to\:{\left[FeH\right]}_{ads}^{+}\:$$

$$\:{\left[FeH\right]}_{ads}^{+}+{\:\text{e}}^{-}\:\to\:\:{\left[FeH\right]}_{ads}$$

$$\:{\left[FeH\right]}_{ads}+\:{\text{H}}^{+}+{\:\text{e}}^{-}\to\:\text{F}\text{e}+\:{\text{H}}_{2}$$

**Overall reaction**

$$\:{\text{F}\text{e}}_{\left(\text{s}\text{o}\text{l}\text{i}\text{d}\right)}+{2\text{H}}^{+}\to\:{\text{F}\text{e}}^{2+}+{{\text{H}}_{2}}_{\left(\text{g}\text{a}\text{s}\right)}$$

The following equation represents the influence of both inhibitors on the mechanism of corrosion of steel in hydrochloric acid^130^.

**Adsorption on a steel surface**

$$\:\text{F}\text{e}+TAC\iff\:{[\text{F}\text{e}.\text{T}\text{A}\text{C}]}_{\text{a}\text{d}\text{s}}$$

**Formation of protective film**

$$\:{\text{F}\text{e}}^{2+}+TAC\iff\:{[{\text{F}\text{e}}^{2+}.\text{T}\text{A}\text{C}]}_{\text{a}\text{d}\text{s}}$$

**Reducing the rate of anodic reaction**

$$\:{\text{F}\text{e}}^{2+}+TAC\:+\:{\text{C}\text{l}}^{-}\iff\:{[FeCl.\text{T}\text{A}\text{C}]}_{\text{a}\text{d}\text{s}}$$

**Reducing the rate of cathodic reaction**

$$\:{[FeH.TAC]}_{ads}+\:{\:\text{e}}^{-}\to\:\text{F}\text{e}+\:{\text{H}}_{2}+{TAC}_{dersorbed}$$

**Overall corrosion inhibition reaction**

$$\:{\text{F}\text{e}}_{\left(\text{s}\text{o}\text{l}\text{i}\text{d}\right)}+{2\text{H}}^{+}+{\text{T}\text{A}\text{C}}_{ads.}\to\:{\text{F}\text{e}}^{2+}+{{\text{H}}_{2}}_{\left(\text{g}\text{a}\text{s}\right)}+{\text{T}\text{A}\text{C}}_{desorbed}$$

By the addition of different inorganic salts, the corrosion inhibition efficiencies increased through the addition of cooperative adsorption which is represented in the following equations for the addition of copper chloride salt.

**Cu**^**2+**^ **Reduction and Deposition on the Steel Surface**

$$\:{\text{C}\text{u}}^{2+}+{2\text{e}}^{-}\to\:{\text{C}\text{u}}_{\text{a}\text{d}\text{s}\text{}}$$

**Complex Formation with the Inhibitor**

$$\:{\text{C}\text{u}}_{\text{a}\text{d}\text{s}\text{}}+\text{T}\text{A}\text{C}\rightleftharpoons\:{[\text{C}\text{u}.\text{T}\text{A}\text{C}]}_{\text{a}\text{d}\text{s}}\text{}$$

**Anodic Reaction (Blocking Active Sites)**

$$\:{\text{F}\text{e}}^{2+}+{\text{C}\text{u}\text{C}\text{l}}_{2}\text{}+\text{T}\text{A}\text{C}\rightleftharpoons\:{[\text{F}\text{e}\text{C}\text{u}\text{C}\text{l}.\text{T}\text{A}\text{C}]}_{\text{a}\text{d}\text{s}}\text{}$$

**Cathodic Reaction (Interference with Hydrogen Evolution)**

$$\:{[\text{F}\text{e}\text{H}.\text{T}\text{A}\text{C}]}_{\text{a}\text{d}\text{s}}\text{}+{\text{C}\text{u}}^{2+}\to\:\text{F}\text{e}+{\text{C}\text{u}}_{\text{a}\text{d}\text{s}}\text{}+{\text{H}}_{2}\text{}+{\text{T}\text{A}\text{C}}_{\text{d}\text{e}\text{s}\text{o}\text{r}\text{b}\text{e}\text{d}}\text{}$$

## Conclusions

From the results, we can conclude:

1. The examined cationic Gemini surfactants (TAC 6 and TAC 18) demonstrated high efficiency in protecting carbon steel from corrosion in 1 M HCl.
2. The inhibition efficiency increases with inhibitor concentration, reaching its maximum at 50 ppm.
3. The inhibition efficiency follows the order TAC 18 > TAC 6, highlighting the greater protective effect of longer hydrocarbon chains.
4. Higher temperatures (303 K to 323 K) reduce inhibition efficiency, suggesting a weaker inhibitor-surface interaction at elevated temperatures.
5. The inhibitors TAC 6 and TAC 18 adsorbed onto the steel surface following Langmuir’s isotherm, indicating monolayer adsorption.
6. Kinetic and thermodynamic parameters confirm the presence of both physisorption and chemisorption, contributing to the protective film formation.
7. The shift in corrosion potential confirms that TAC 6 and TAC 18 act as cathodic inhibitors, primarily reducing hydrogen evolution.
8. Over time, pores form in the protective layer on the steel surface. Still, their occurrence decreases with higher inhibitor concentration, leading to a denser, more uniform film and improved corrosion resistance.
9. The theoretical calculation using quantum DFT confirms the same conclusions indicated by chemical and electrochemical techniques.
10. The addition of inorganic salts enhances inhibition efficiency, following the order: CuCl₂ > MnCl₂ > CoCl₂.
11. The cationic portion of the salts co-adsorbs alongside the inhibitor molecules, forming a more stable and uniform protective layer in the order: Cu²⁺ > Mn²⁺ > Co²⁺.
12. In the presence of salts, TAC 6 and TAC 18 shift from dominant cathodic inhibitors to mixed-type inhibitors, effectively suppressing both anodic and cathodic corrosion reactions.

## Data Availability

Raw data were generated at Faculty of Science, Port-Said University, Egypt. Derived data supporting the findings of this study are available from the corresponding author, Prof. Dr. Samir A. Abd El-Maksoud, on request.
